# Steep Glacier Bed Knickpoints Mitigate Inland Thinning in Greenland

**DOI:** 10.1029/2020GL090112

**Published:** 2021-01-19

**Authors:** Denis Felikson, Ginny A. Catania, Timothy C. Bartholomaus, Mathieu Morlighem, Brice P. Y. Noël

**Affiliations:** ^1^ Cryospheric Sciences Laboratory NASA Goddard Space Flight Center Greenbelt MD USA; ^2^ Goddard Earth Sciences Technology and Research Studies and Investigations Universities Space Research Association Columbia MD USA; ^3^ University of Texas Institute for Geophysics University of Texas at Austin Austin TX USA; ^4^ Department of Geological Sciences University of Texas at Austin Austin TX USA; ^5^ Department of Geological Sciences University of Idaho Moscow ID USA; ^6^ Department of Earth System Science University of California, Irvine Irvine CA USA; ^7^ Institute for Marine and Atmospheric Research Utrecht Utrecht University Utrecht The Netherlands

## Abstract

Greenland’s outlet glaciers have been a leading source of mass loss and accompanying sea‐level rise from the Greenland Ice Sheet (GrIS) over the last 25 years. The dynamic component of outlet glacier mass loss depends on both the ice flux through the terminus and the inland extent of glacier thinning, initiated at the ice‐ocean interface. Here, we find limits to the inland spread of thinning that initiates at glacier termini for 141 ocean‐terminating outlet glaciers around the GrIS. Inland diffusion of thinning is limited by steep reaches of bed topography that we call “knickpoints.” We show that knickpoints exist beneath the majority of outlet glaciers but they are less steep in regions of gentle bed topography, giving glaciers in gentle bed topography the potential to contribute to ongoing and future mass loss from the GrIS by allowing the diffusion of thinning far into the ice sheet interior.

## Key Points

- Greenland glaciers with high potential to contribute to mass loss identified using novel metric
- The majority of thinning limits are set by a knickpoint, a steep drop in bed topography
- Glaciers that transmit thinning far inland are preferentially in regions with gentle bed topography

## 1 Introduction

Since the early‐ to mid‐1990s, the Greenland Ice Sheet (GrIS) has been losing mass at an accelerating rate, with dynamic mass loss due to increased discharge from ocean‐terminating outlet glaciers accounting for 66 ± 8% of total mass loss (Mouginot et al., 2019). Retreat and acceleration of outlet glaciers (Bevan et al., 2012; Holland et al., 2008; Khan et al., 2014; Kjær et al., 2012; Larsen et al., 2016) has caused thinning, initiated at outlet glacier termini, to diffuse inland (Bindschadler, 1997; Nick et al., 2009; Nye, 1960; Payne et al., 2004; Price et al., 2011; van der Veen, 2001). Over the long‐term (>3 years), the diffusion of thinning can account for >75% of committed ice sheet mass loss over the century following initial terminus retreat (Price et al., 2011). Past studies have examined how glacier geometry controls terminus retreat (Carr et al., 2014; Catania et al., 2018; Gudmundsson et al., 2012 Jamieson et al., 2012, 2014; Robel et al., 2016; Schoof, 2007; Steiger et al., 2018) and how glacier geometry influences thinning near the terminus (McFadden et al., 2011). However, there has not been a study to assess the potential for diffusive thinning further inland beyond the near‐terminus region, over the entirety of the GrIS, a topic of critical importance for centennial sea‐level rise estimates (Price et al., 2011).

Recent work found that thinning across 13 west Greenland ocean‐terminating outlet glaciers diffuses from the terminus over some distance until further diffusion is impeded by the unique geometry of each glacier (Felikson et al., 2017, here referred to as F17). The along‐flow locations beyond which the upstream diffusion of thinning is limited are called “thinning limits.” We expand on F17 by examining 141 ocean‐terminating glaciers around the entire GrIS and examining the geometric features that limit the inland diffusion of thinning. Further, we improve upon the analysis of F17 by surveying glacier geometry across flow and applying a more rigorous uncertainty quantification. This allows us to identify upstream limits to diffusive thinning for all 141 glaciers, even if they have not yet thinned. We find that most thinning limits are proximal to the location where the subglacial bed topography transitions from the submarine trough, below sea level, to the inland bed, above sea level. Closer examination of the bed topography in these regions allows us to identify steep step changes in bed topography (>300 m over 100 km or less) that we call “knickpoints” and we examine their geometries in differing regional bed topographic settings. Finally, we use the distances from the ice margin to inland thinning limits and each glacier’s ice flux to examine each glacier’s potential for dynamic ice mass loss in response to terminus perturbations.

## 2 Methods

Along glacier flow, thinning perturbations that are initiated by terminus retreat evolve as a diffusive‐kinematic wave, advecting downstream with flow and diffusing both upstream and downstream (Nye, 1960). The ratio of the rates of downstream advection to diffusion of the thinning wave is expressed by the Péclet number (Pe). Where Pe is high along glacier flow, the rate of downstream advection of thinning will outpace upstream diffusion; thus, by finding locations of high Pe, we can identify the upstream limits to dynamic thinning (Felikson et al., 2017). The diffusive‐kinematic wave formulation arises from a linearized perturbation of the one‐dimensional mass transport equation and assumes that the stress balance of the glacier is local, neglecting longitudinal stress coupling. Using an idealized model, Williams et al. (2012) found that, in the limit of low frequency forcing, perturbations propagate upstream through changes in glacier geometry rather than through direct transmission of longitudinal stresses. This provides rationale for using the diffusive‐kinematic wave approach to study glacier response to low frequency forcing. Nevertheless, we account for longitudinal stress coupling and reduce random error by smoothing glacier ice surface and bed elevations within windows of 10 local ice thicknesses at 50‐m intervals along glacier flow (Kamb & Echelmeyer, 1986).

The coefficients governing the rates of advection and diffusion in the diffusive‐kinematic wave equation are partial derivatives of ice flux with respect to thickness and surface slope, respectively. To calculate these coefficients, we relate glacier geometry to ice flux by assuming that the ice flux is governed by sliding at the ice‐bed interface (Weertman, 1957). For ice geometry, we use BedMachine (version 3) bed topography (Morlighem et al., 2017) and a digital elevation model (DEM) of the ice sheet surface acquired between 1978 and 1987 (Korsgaard et al., 2016). Beyond the spatial extent of the 1978–1987 DEM, we use surface topography from the Greenland Ice Mapping Project (GIMP; Howat et al., 2014). Under these assumptions, we calculate Pe as:

(1) $$Pe\left(x\right)=2\;\frac{b^{\prime }\left(x\right)}{H_{0}\left(x\right)}\;l\left(x\right)$$

where *H*_0_ is the smoothed glacier thickness, and *b*′ is the smoothed bed slope along flow. In our formulation, *x* points in the upstream direction. The length scale, *l*, represents the length of the perturbation and here we assume that the perturbation extends from the terminus to each location, *x*, along flow (thus, *l*(*x*) = *x*). Negative values of Pe represent locations along glacier flow where thinning perturbations can advect upstream and, because of the particular flux‐geometry relationship that we use, this occurs where the bed slope is negative (i.e., becoming deeper in the upstream direction). Previous work has shown that using other typical formulations for the relationship between ice flux and geometry does not significantly affect the locations of our predicted flowline thinning limits (Felikson et al., 2017). Furthermore, in this study, we are focused on finding locations where Pe is positive and large enough to prevent upstream diffusion and, thus, we do not elaborate any further on negative Pe values. Additional information about the derivation of Pe can be found in Text S3 in the supporting information.

For each outlet glacier, we measure total thinning by differencing the 1978–1987 DEM from a DEM of the ∼2015 ice sheet surface elevation (Porter et al., 2018). We calculate dynamic thinning by removing the surface mass balance (SMB) anomaly, integrated over the ∼35 year period, from total thinning using the regional climate model RACMO2.3p2, downscaled to 1 km (Noël et al., 2019, Figure S1 in the supporting information). We create a comprehensive list of ocean‐terminating glaciers from two previous catalogs (I. Joughin et al., 2015, 2017; Moon & Joughin, 2008; Rignot & Mouginot, 2012) and identify ocean‐terminating glaciers that have well‐constrained bed topography in the BedMachine product (Morlighem et al., 2017). Of these 141 glaciers, 34 have dynamic thinning that extends from their termini to the inland edge of the DEM difference and, thus, the spatial extent of dynamic thinning cannot be measured using our method (Figure S1 in the supporting information). Another 35 glaciers have not dynamically thinned, leaving 72 glaciers that have dynamic thinning limited to within the spatial extent of the DEM difference. We use dynamic thinning observations of these 72 glaciers to find an empirical limit to the diffusion of thinning following the method of F17 and find that 89% of observed total cumulative thinning along flowlines (interquartile range of 74%–100%) occurs downstream of where Pe first exceeds 3, within the uncertainty bounds found by F17. See Text S4 and Figure S3 in the supporting information for more information on the calibration of the empirical thinning limit. In a predictive sense, we expect thinning to diffuse from the terminus to the location where Pe first exceeds 3 for all glaciers, irrespective of their having thinned previously, and we use this “predicted flowline thinning limit” to identify the inland extent of thinning for all 141 glaciers, including those that have not yet thinned. If Pe does not exceed 3 along the entire length of a flowline, we assume that thinning can diffuse from the terminus to the ice divide and we set the predicted flowline thinning limit to be at the upstream end of the flowline at the divide.

We provide two improvements to the previous analysis of F17. First, we assess each glacier’s across‐flow geometry with 6–24 flowlines, allowing us to account for heterogeneity in the across‐flow geometry of each glacier. For each of the 141 glaciers, we find the furthest inland predicted flowline thinning limit through an iterative approach using increasingly finer across‐glacier resolution and a variable number of flowlines (Text S1 in the supporting information). We then create one measure of the predicted inland thinning distance for each glacier, taking into consideration both (1) the furthest inland predicted flowline thinning limit, which accounts for the worst‐case, furthest‐inland distance over which thinning can diffuse, and (2) the spread in distances to predicted flowline thinning limits across glacier flow, which accounts for our uncertainty in the extent of thinning. We call this the “predicted glacier thinning limit,” calculated by subtracting one standard deviation of the distances to all predicted flowline thinning limits from the distance to the single furthest inland predicted flowline thinning limit. Herein, we discuss thinning limits along individual flowlines (predicted flowline thinning limits), which are used to examine spatial patterns of thinning within individual glaciers, as well as predicted glacier thinning limits, which are used to compare each glacier to one another.

As an additional improvement on F17, we quantify uncertainties in both observed dynamic thinning and Pe and propagate these through our calculations of predicted glacier thinning limits. We shift values of dynamic thinning and Pe along each flowline using a systematic bias obtained from reported uncertainties in bed topography, surface DEMs, and RACMO‐estimated SMB to obtain worst‐case bounds on our calculated values of dynamic thinning and Pe (Text S9 and S10 in the supporting information). Using a Monte Carlo analysis, we randomly sample from these worst‐case calculations to examine uncertainty in our calibration of an empirical thinning limit and in the glacier thinning limits (detailed description of uncertainty analysis in Text S11 and S12 in the supporting information). We find that, even in this conservative uncertainty analysis, statistically, the majority of dynamic volume loss occurs downstream of Pe = 3 (Figures S8 and S9 in the supporting information). Additionally, because our chosen value for the empirical thinning limit is somewhat arbitrary, we examine the effect of this choice on our results by considering Pe = 2 and Pe = 4 as thinning limit locations and discuss the implications of that choice on our results. Finally, while we expect that Pe values will change as glacier geometry evolves, we have found that observed changes in Pe are not large even in the presence of the dramatic dynamic thinning observed over the last 30 years (Felikson et al., 2017). Thus, we argue that Pe is a useful predictor for the region over which dynamic thinning will occur over the coming decades to century.

## 3 Results and Discussion

**Figure 1:**
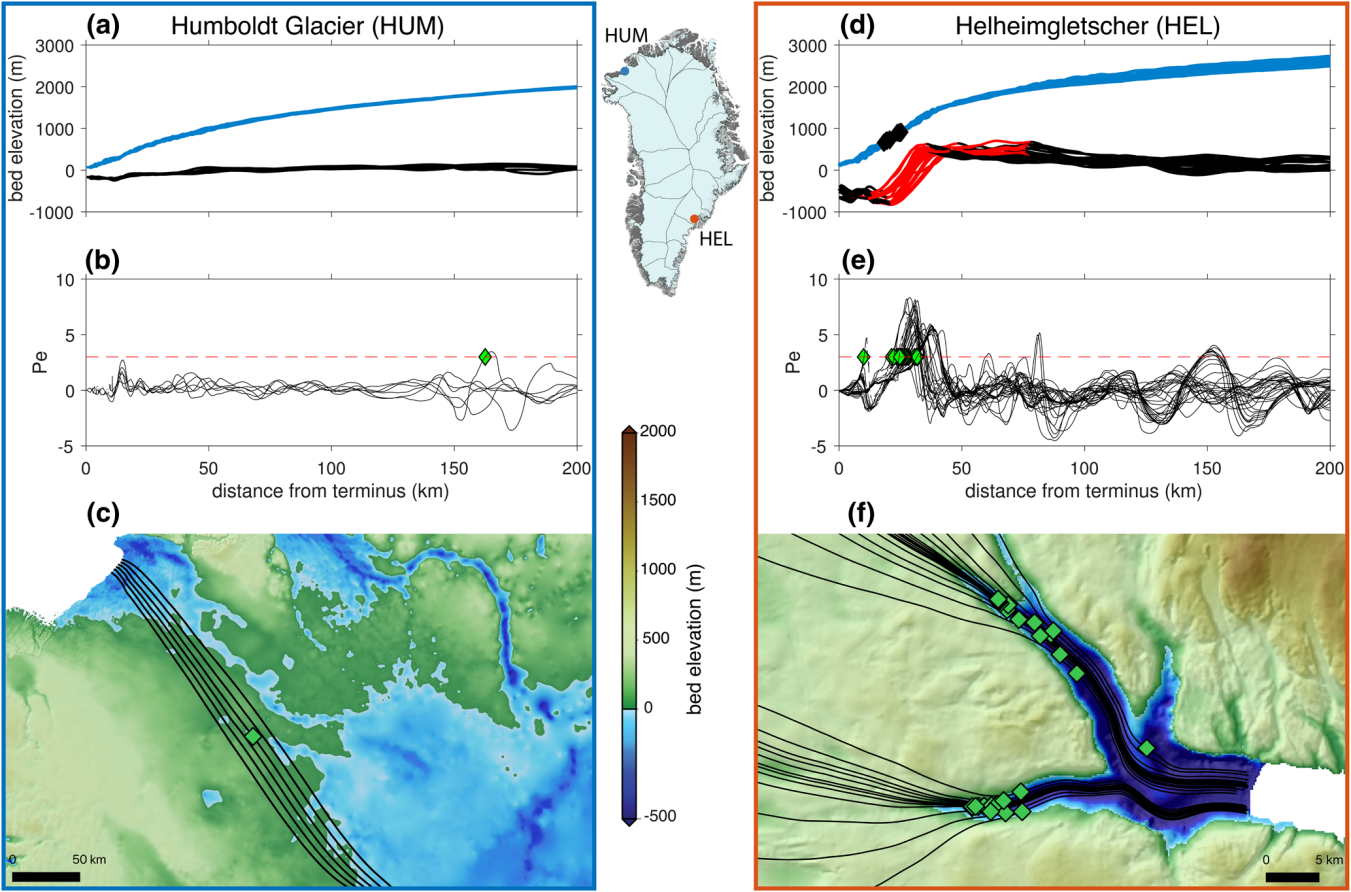
Geometry and predicted flowline thinning limits for two GrIS outlet glaciers. (a) Smoothed topography along Humboldt Gletscher flowlines with distance from terminus showing bed (black) and ice sheet surface (blue) along surveyed flowlines. (b) Péclet number along flowlines (gray) and Pe = 3 threshold (dashed red line). (c) Flowlines overlaid on top of bed topography in map view. (d)**–**(f) Similar to (a–c) at Helheimgletscher; identified knickpoints (red) in (d); locations where observed cumulative thinning along flowlines reaches 89% are shown as black diamonds in (d) (further discussed in the text); predicted flowline thinning limits shown as green diamonds in (d and f). Green diamonds in (b, c, e, and f) show locations of predicted flowline thinning limits. Glacier locations shown on GrIS map insert.

Examining predicted flowline thinning limits, we can categorize the results according to two end‐member glacier geometries: (1) those that permit thinning perturbations to diffuse very far inland and (2) those that set strong limits to the inland diffusion of thinning. We demonstrate the differences between these two geometry types through examination of two representative examples. Humboldt Gletscher (HUM) in Northwest Greenland flows over gentle bed topography from the ice sheet interior to its submarine terminus (Figure 1a). The Pe along all flowlines for HUM remains low and never exceeds the empirical limit to thinning (Pe = 3), thereby indicating that thinning that originates at the terminus of this glacier will be able to diffuse far into the interior of the ice sheet (Figure 1b). Conversely, for Helheimgletscher (HEL) in East Greenland, inland flow converges over steep steps in the bed that separate the inland bed from the submarine troughs of the fast‐flowing glacier trunk (Figure 1d). Using our automated knickpoint detection algorithm (Text S7 in the supporting information), we find that all HEL flowlines have knickpoints (red highlights in Figure 1d) whereas none of the HUM flowlines have identifiable knickpoints because the bed slope for this glacier is gentle (Figure 1a). At the HEL knickpoints, steeper surface slopes (2.8°) and relatively thinner ice lead to higher Pe values (Pe > 3) than anywhere else along the glacier (Figure 1). The steep slopes of the HEL knickpoints and the fact that they are all co‐located provides a strong control that limits the inland diffusion of thinning. Indeed, the locations where cumulative thinning along HEL flowlines reaches 89% of total cumulative thinning is within the extent of the knickpoints, thus coinciding with the predicted flowline thinning limits for this glacier (black diamonds in Figure 1d).

**Figure 2:**
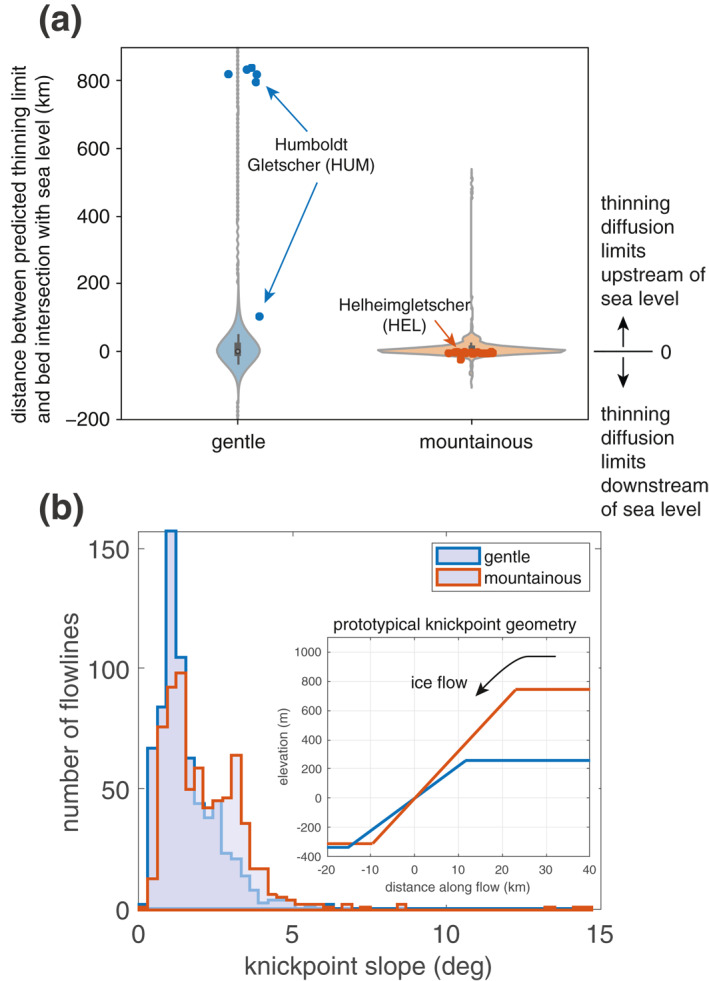
Distances from GrIS margin to predicted flowline thinning limits and knickpoint slopes in regions of gentle and mountainous bed topography. (a) Violin plots of distances between predicted flowline thinning limits and locations (along flow) where the bed rises above sea level for all GrIS flowlines within regions of gentle and mountainous bed topography (Helheimgletscher distances shown as red dots; Humboldt Gletscher distances shown as blue dots). Violin plots show box and whisker plot as well as a kernel density estimation of the underlying distribution of data (i.e., the width of the shaded area represents the relative proportion of the number of flowlines located at a particular distance). Thinning limits that are collocated exactly with the location where the bed rises above sea level would plot along *y* = 0. Predicted flowline thinning limits that are upstream of the location where the bed rises above sea level plot above *y* = 0 and those that are downstream plot below *y* = 0. (b) Normalized histograms of the slopes of identified knickpoints at the location where the bed rises above sea level. Inset shows a prototypical knickpoint using the median trough depths, median knickpoint slope, and median upstream height for knickpoints in gentle regions (blue) and mountainous regions (red).

Knickpoints are prevalent beneath GrIS outlet glaciers and play an important role in limiting the inland diffusion of thinning. Sixty‐eight percent of all surveyed flowlines have an identified knickpoint. To ensure that our sampling of glaciers with a variable number of flowlines does not skew this statistic, we survey one centerline per glacier and find that 65% of glacier centerlines have an identified knickpoint. Of those flowlines with an identified knickpoint, 88% have predicted flowline thinning limits within the extent of the knickpoints, indicating that knickpoints are important geometric features for mitigating the inland diffusion of terminus‐initiated thinning for the majority of outlet glaciers in Greenland. Knickpoints have a median height of hundreds of meters, relative to the median elevation of the trough, spatial extent of tens of kilometers (inset in Figure 2b), and are located at heterogeneous distances, from 1.3 to 50 km, inland from glacier termini.

Around the GrIS, we find that the regional topographic setting influences the presence, location, and geometry of knickpoints and, thus, the associated predicted flowline thinning limits for outlet glaciers. When we separate the ice sheet into regions of mountainous and gentle bed topography (Text S6 in the supporting information), we find that the predicted flowline thinning limits that lie furthest inland are in regions with gentle bed topography (Figure 2a). Conversely, in mountainous terrain, the median knickpoint slope is 46% steeper than in gentle topography, with statistical significance at the 1% level (Figure 2b). One hypothesis to explain these differences is due to topographic steering of ice flow through mountainous topography (Kessler et al., 2008). The combination of flow confluence within glacier tributaries (Kessler et al., 2008; MacGregor et al., 2000) and higher basal sliding speeds within glacier troughs produces higher erosion rates in glacier trunks compared to the slow‐flowing interior ice (Hallet, 1979; Herman et al., 2015). Modeling has shown that steeper knickpoints are notched into the landscape where ice is topographically steered through high relief (Kessler et al., 2008). Our observations corroborate this model expectation; flowlines for glaciers situated in mountainous topography converge to flow down relatively steeper knickpoints (Figures 1f and 2b), causing more flowlines within a glacier to have predicted flowline thinning limits that are set by knickpoints. Thus, knickpoints in mountainous topography exert a strong control on the diffusion of thinning because all flowline thinning limits are collocated at the knickpoint. In contrast, glaciers in gentle topographic settings have flowlines that are not strongly topographically steered to converge. Thus, steep knickpoints are not formed as easily in these locations, and we find larger discrepancies between the locations of predicted flowline thinning limits and where the bed rises above sea level, often with predicted flowline thinning limits located far inland along multiple flowlines (Figure 2a). Thus, because there is more heterogeneity in the presence of knickpoints or their slope along flowlines of glaciers in gentle bed topography, knickpoints exert weak control on the diffusion of thinning in regions of gentle bed topography.

**Figure 3:**
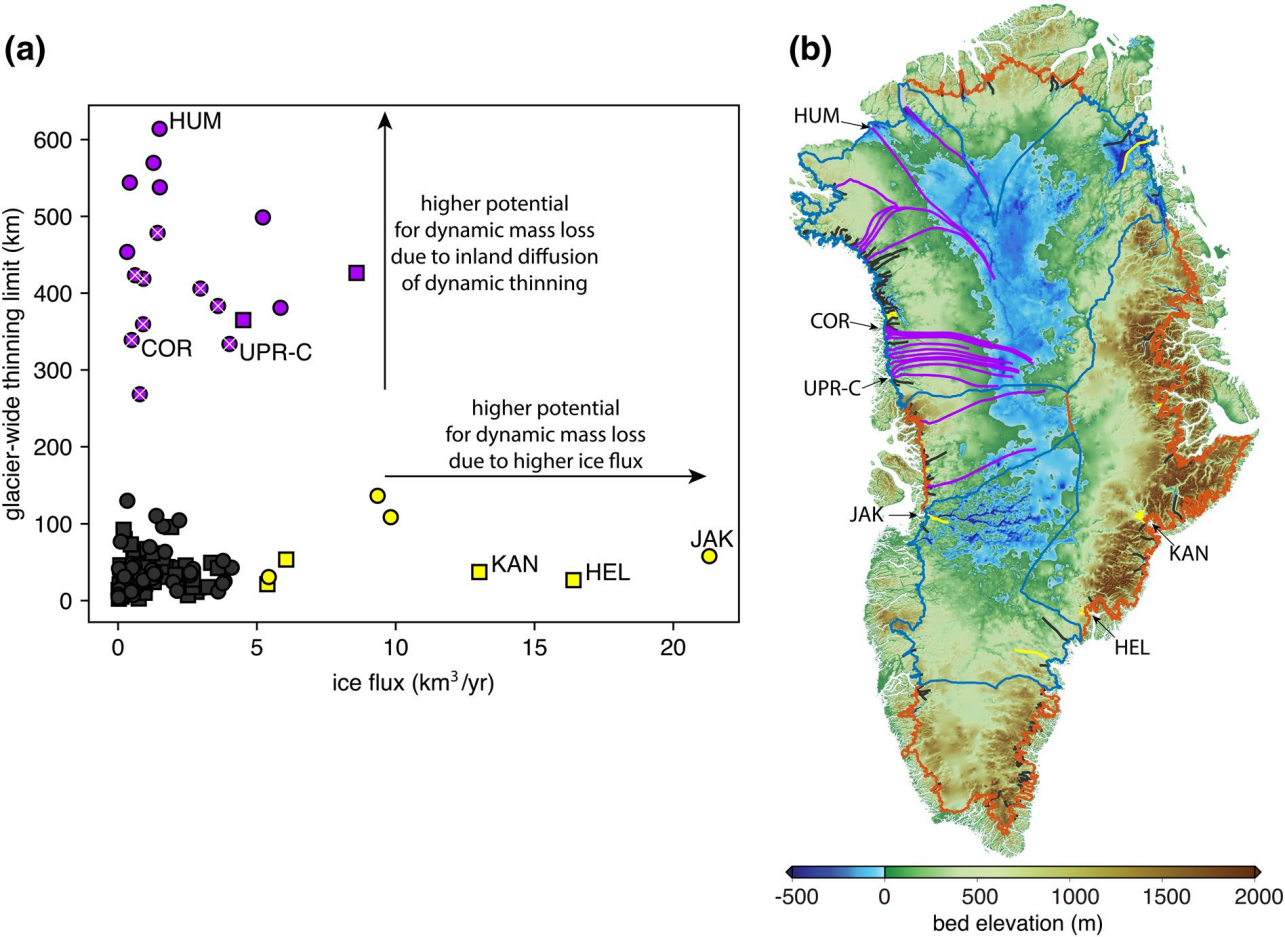
Glacier thinning limits and potential for dynamic mass loss of 141 GrIS outlet glaciers. (a) Distances from ice sheet margin to thinning limits plotted against ice fluxes for glaciers in regions of gentle (circles) and mountainous (squares) bed topography. Purple markers indicate a group of glaciers with thinning limits >200 km from the ice margin; yellow markers indicate a group of glaciers with >5 km^3^/yr ice flux. White x's inside purple markers indicate nine glaciers in NW Greenland, discussed in the text. (b) Flowlines for each glacier drawn from the terminus to the predicted glacier thinning limit and colored according to groupings shown in (a), shown on top of Greenland bed topography. Regions of mountainous bed topography (red coastlines) and gentle bed topography (blue coastlines) shown. Upernavik Isstrøm C (UPR‐C), Cornell Gletscher (COR), Humboldt Gletcher (HUM), Kangerlussuaq Gletscher (KAN), Helheimgletscher (HEL), and Jakobshavn Isbræ (JAK) referenced in the text, are labeled in both panels.

A glacier’s potential for dynamic ice mass loss is governed by both the inland extent of thinning resulting from terminus retreat and the glacier’s ice flux. A glacier with a far inland thinning limit has the potential for large dynamic mass loss because thinning can draw down a larger part of the ice sheet, whereas a glacier with high ice flux has the potential for large dynamic mass loss because a perturbation to its flux can lead to larger ice discharge than a glacier with small ice flux. In other words, a 1% perturbation to the flux of a high ice flux glacier yields larger discharge and, thus, more dynamic mass loss than for a low ice flux glacier. Figure 3a shows glacier thinning limits plotted against ice flux, revealing two distinct groups of glaciers with a high potential for dynamic mass loss for two distinct reasons. The first is a group of glaciers with relatively high ice flux (>5 km^3^/yr) but with thinning limits close to the ice sheet margin (<150 km). These glaciers include the well‐studied glaciers Jakobshavn Isbræ (JAK), Kangerlussuaq Gletscher (KAN), and Helheimgletscher (HEL), which are often examined in the literature due to their high flux. A second group of glaciers exists with relatively low ice flux (<10 km^3^/yr) but far inland thinning limits (>200 km). These glaciers do not receive much focused attention in the literature but they have the potential to be large contributors to dynamic ice mass loss as thinning perturbations diffuse hundreds of kilometers into the ice sheet interior within their catchments. The glaciers in this group are predominantly located in regions of gentle bed topography (circles in Figure 3a; blue outlines in Figure 3b), where we have shown that knickpoints tend to be less steep (Figure 2b), allowing the potential for thinning to diffuse further inland (Figure 2a), and causing glaciers to have a high potential for future dynamic mass loss. The rate of diffusion of thinning will slow as thinning spreads into the ice sheet interior (van der Veen, 2001) and, thus, glaciers in gentle bed topography with far inland thinning limits will continue to thin on a longer timescale than glaciers in mountainous topography, whose thinning limits are located close to the ice sheet margin. By evaluating each glacier’s potential contribution, we are examining the relative response of each glacier to an equal perturbation at the front. The actual contribution of each glacier to future dynamic ice‐sheet mass loss will depend on the magnitude of a perturbation at its terminus, which is something we do not explicitly take into account.

Of particular concern are a group of glaciers in the gentle topography of Northwest Greenland, between Upernavik Isstrøm C (UPR‐C) and Cornell Gletscher (COR), where 9 of 12 neighboring glaciers have predicted glacier thinning limits >250 km from the ice sheet margin and, thus, they can collectively draw down a ∼140‐km‐wide region far into the ice sheet interior. By considering uncertainties in input datasets and our choice of Pe = 3 as the empirical thinning limit, we find that predicted glacier thinning limits are most uncertain in regions of gentle bed topography around the GrIS (Figure S10 in the supporting information). In contrast, locations of predicted glacier thinning limits in mountainous regions are more certain because knickpoints in these locations are steep enough and tall enough that they tend to set strong geometric control on thinning and persist even in our most conservative uncertainty estimates. Because of the higher uncertainty in the locations of their thinning limits, glaciers in gentle topography may thin farther into the interior than predicted by our nominal predicted flowline thinning limit locations, highlighting their importance for further study. We find no glaciers with both a high ice flux and far inland thinning limits (i.e., upper‐right of Figure 3a) and we hypothesize that this is because glaciers self‐regulate through erosion of the bed. High‐flux glaciers have more erosive power and create knickpoints that set thinning limits closer to the ice margin where ice flow converges enough to create substantial ice flux.

Our results provide a mechanistic understanding for satellite observations that have measured the largest rates of dynamic thinning and mass loss along the southeast and northwest margins (Csatho et al., 2014; Velicogna et al., 2014). Northwest Greenland is the only region in which ice discharge is experiencing an ongoing, sustained increase since the mid‐2000s (King et al., 2020). We show that glaciers in the gentle topography of the northwest can allow thinning to diffuse far inland, causing glaciers in this region to respond slowly but steadily to terminus perturbations. Thus, we expect discharge in the northwest to continue to remain elevated as thinning continues to diffuse into the interior. In contrast, most glaciers in the mountainous southeast (e.g., Helheimgletcher and Kangerlussuaq Gletscher) have near‐coastal predicted thinning limits that will impede thinning from diffusing far inland. Thus, these glaciers can experience large dynamic mass losses as a result of their high ice flux, however we expect them to restabilize more quickly to terminus perturbations. Furthermore, the predicted inland thinning limits that we have identified can be used to interpret observed ice sheet changes; dynamic thinning inland of our predicted thinning limits is more likely to occur due to processes other than terminus retreat, such as an increase in basal sliding due to excess surface melt or due to subglacial lake drainage.

Although knickpoints may have a different initiation mechanism than their riverine counterparts, by drawing on ideas from fluvial geomorphology, subglacial knickpoints may also be used to reconstruct uplift rate histories (Roberts & White, 2010) and analyze the reorganization of ice drainage basins (Willett et al., 2014), as has been done for fluvial knickpoints. Through the erosion of knickpoints, Greenland’s past geologic activity has implications for modern and future ice sheet mass balance. The formation of mountains in East Greenland 350 Myr ago (McKerrow et al., 2000) produced the bed topography that ultimately controls contemporary inland dynamic mass loss. If we assume that erosion rates within submarine troughs are between 1 and 5 mm/yr (Koppes & Montgomery, 2009), and erosion rates in the ice sheet interior are insignificant, then a 500 m tall knickpoint could have formed over 100,000–500,000 years. Therefore, knickpoints must be slowly evolving features that have likely persisted over multiple past glacial cycles. In this manner, mountain building, glacier dynamics, and geomorphology combine around the ice sheet margins to produce persistent landscape features that exert a stabilizing influence on modern ice sheet mass balance. This highlights the need to include knickpoint evolution in long‐timescale ice sheet models, in order to capture their stabilizing effects on ice dynamics during interglacial periods.

## 4 Conclusion

Using a novel metric easily calculable from glacier geometry, we predict the inland extent of thinning for 141 GrIS outlet glaciers. This metric quantifies the ratio of the rate of advection to the rate of diffusion of a kinematic wave of thinning initiated at glacier termini. We find that the majority of limits on the diffusion of thinning are set by steep knickpoints in subglacial bed topography at the heads of troughs where the bed transitions from below to above sea level. Knickpoints at the heads of subglacial troughs are the locations where we would expect a large decrease in ice discharge when, sometime in the future, glacier retreat causes marine‐terminating outlets to become land‐terminating. We have shown that these same locations are already mitigating the impact of marine‐terminating outlet glaciers on dynamic ice sheet mass loss by limiting the diffusion of thinning further inland. Consequently, we find that glaciers that allow thinning to diffuse far into the interior of the GrIS are preferentially located in regions of gentle bed topography because, although knickpoints are prevalent around the entire ice sheet, they are less steep in regions of gentle bed topography. When we consider both inland thinning limits and ice flux as measures of each glacier’s potential for dynamic mass loss, we find that glaciers with low ice flux may be significant contributors because of their ability to transmit thinning far inland. We hypothesize that the low ice flux glaciers with far inland thinning limits will contribute to dynamic ice sheet mass loss over a longer timescale than high ice flux glaciers, as the rate of diffusion of thinning slows in the ice sheet interior. Of particular concern is a group of glaciers along the northwest coast of the GrIS, between Upernavik Isstrøm C and Cornell Gletscher, where 9 of 12 neighboring glaciers have the potential to lead to long‐term, diffusive thinning >250 km into the interior of the ice sheet over a ∼140‐km‐wide region. This provides a mechanistic explanation for why the northwest sector of the GrIS is the only region experiencing an ongoing increase in observed discharge.

## Supporting information

Supporting Information S1

Table S2

## Data Availability

BedMachine, version 3, bed topography is available through the National Snow and Ice Data Center (NSIDC, https://nsidc.org/data/idbmg4). The 1978–1987 DEM is available at the National Oceanic and Atmospheric Administration National Centers for Environmental Information (https://doi.org/10.7289/v56q1v72). Greenland Ice Mapping Project (GIMP) DEM is available through NSIDC (https://nsidc.org/data/nsidc-0645). RACMO2.3p2 surface mass balance provided by B. P. Y. Noël and M. R. van den Broeke. All data, sampled and calculated along glacier flowlines, are available via Zenodo (https://doi.org/10.5281/zenodo.4284759). Scripts to read and manipulate flowline data and reproduce figures in the manuscript are available on Zenodo at: https://doi.org/10.5281/zenodo.4284715.
