# Supplementary material for: Steep Glacier Bed Knickpoints Mitigate Inland Thinning in Greenland
Source: Geophys Res Lett. 2021 Jan 19;48(2):e2020GL090112. doi: 10.1029/2020GL090112 (PMC7900969; doi:10.1029/2020GL090112)
Supplement: Supplementary file 1 — Supporting Information S1 [file GRL-48-e2020GL090112-s001.pdf]

Supporting information for: Steep glacier bed knickpoints mitigate inland thinning in Greenland

Denis Felikson[1], Ginny Catania[2,3], Timothy C. Bartholomaus[4], Mathieu Morlighem[5], Brice P. Y. Noël[6]

[1]Cryospheric Sciences Laboratory, NASA Goddard Space Flight Center, Greenbelt MD, 20771, USA

[2]University of Texas Institute for Geophysics, University of Texas at Austin, Austin, TX 78712, USA

[3]Department of Geological Sciences, University of Texas at Austin, Austin, TX 78712, USA

[4]Department of Geological Sciences, University of Idaho, Moscow, ID 83844, USA

[5]Department of Earth System Science, University of California, Irvine, CA 92697, USA

[6]Institute for Marine and Atmospheric Research Utrecht, Utrecht University, 3508 TA Utrecht, NL

Corresponding author: D. Felikson, Cryospheric Sciences Laboratory, NASA Goddard Space Flight Center 8800 Greenbelt Rd, Greenbelt, MD 20771, USA. (denis.felikson@nasa.gov)

Contents of this file

1. Text S1 to S12
2. Figures S1 to S10
3. Table S1

Additional Supporting Information (Files uploaded separately)

1. Table S2: Glacier information. Table lists glacier id, glacier name, glacier-wide thinning limit distance from terminus, glacier ice flux, knickpoint presence, and knickpoint slope if a knickpoint is present.

Introduction

This supporting information includes text, figures, and tables that provide a detailed description of the methods used in the study and a more thorough interpretation and analysis of the uncertainty, which is summarized in main text. An additional table is provided with glacier-wide thinning limits, ice flux, and knickpoint characteristics for each glacier surveyed in the study.

Text S1. Glacier flowlines

We identified marine-terminating glaciers around the Greenland Ice Sheet by combining glaciers identified in the survey of Rignot and Mouginot (2012) with glaciers identified in the MEaSUREs Annual Greenland Outlet Glacier Terminus Positions from SAR Mosaics, Version 1 dataset (Moon & Joughin, 2008; I. Joughin et al., 2015, 2017). The two datasets define the location of each glacier at approximately the center of flow, a few kilometers from the terminus, and we used the locations defined in each dataset, with the exception of several that were manually moved away from the edge of the ice sheet surface velocity dataset to facilitate creating flowlines.

Rignot and Mouginot (2012) classified 243 land- and marine-terminating glaciers around the Greenland Ice Sheet. We then added 34 glaciers in the MEaSUREs survey that were not included in Rignot and Mouginot (2012). This yielded a total of 277 glaciers. We created centerlines for the 277 glaciers extending up and down glacier flow using the MEaSUREs Multi-year Greenland Ice Sheet Velocity Mosaic, Version 1 dataset. Centerlines were found using Matlab's *stream2* function (I. R. Joughin et al., 2017; I. Joughin et al., 2016) and were clipped to grounded ice using the mask provided with the BedMachine

product (Morlighem et al., 2017; Morlighem, 2017). Because of uncertainty in ice surface velocity field, some centerlines terminated in unending loops. All centerlines were manually inspected and loops were removed.

Along the glacier centerlines, we sampled bed topography along 20 km from each glacier's terminus and we identified ocean-terminating glaciers as those that have >2 km of bed elevation below sea level and whose submarine troughs begin within 1 km of the terminus. Using these criteria, we find 156 ocean-terminating glaciers. Of the 156, 145 have bed topography estimated from mass-conservation in BedMachine. Of the 145 ocean-terminating glaciers with mass-conserving bed topography, we identified 4 glaciers with poor constraints on the mass-conserving bed solution. We identified these poorly-constrained glaciers as ones with either no bathymetry nor radar sounding measurements along their flow or ones with negative ice thickness found from the ArcticDEM and BedMachine bed. Thus, 141 ocean-terminating glaciers with well-constrained bed topography remained and these are the ones that we surveyed in the study.

total	277
ocean-terminating	156
ocean-terminating, mass-conserving bed	145
ocean-terminating, mass-conserving bed, well-constrained	141

Additionally, we manually defined the locations of 75 total tributaries at approximately the center of flow within the tributary, where ice flow is distinct from the main trunk. A centerline and separate across-flowlines were created for each tributary, but when we report glacier-wide statistics, we use flowlines across all tributaries.

Several prior studies have catalogued Greenland ocean-terminating outlet glaciers. (Murray et al., 2015) found 199 ocean-terminating glaciers by identifying glaciers wider

than 1 km using Landsat-7 imagery from 2000-2010. (Howat & Eddy, 2011) employed the same method, identifying glaciers wider than 1 km in satellite imagery, but used additional data from Landsat-1 (1972) through Landsat-7 (2010) to identify 210 ocean-terminating glaciers. (Rignot & Mouginot, 2012) identified 212 ocean-terminating glaciers by compiling a thorough review of previous literature. Our estimate of 156 ocean-terminating outlet glaciers is lower than previous estimates for two reasons. First, some of the ocean-terminating glaciers identified in older studies may have retreated out of the ocean and now terminate on land. Second, uncertainty in the bed topography may cause us to characterize a glacier as land-terminating when, in reality, it terminates in the ocean. Our study is the first to identify ocean-terminating glaciers using the BedMachine, v3, bed topography to find glaciers grounded below sea level. So, while our method may not provide a definitive catalog of all ocean-terminating outlet glaciers, it does provide us with a nearly complete sample with many representative bed topographies that is sufficient for our characterization of the propagation of thinning.

To sample glaciers across-flow, streamlines were created from evenly-spaced starting points across each glacier's flow. Starting points were identified along a line perpendicular to the surface velocity field starting at each glacier's location. The perpendicular line was terminated where surface velocity is less than 60% of the surface velocity at the glacier location. Ten starting points for the across-flow streamlines were placed at evenly-spaced intervals along each glacier's perpendicular line and the two starting points on each extreme of the perpendicular line were discarded because these were often within shear margins. Thus, six across-flow streamlines were created for each glacier in the same

manner as the centerlines, masking to grounded ice, and manually removing unending loops. Additionally, we manually removed one across-flowline from one glacier because it followed the flow of a neighboring tributary.

To account for differences in glacier geometry and, hence, thinning diffusion limits, across glacier flow, we use an iterative approach, finding thinning limits along flowlines across the width of each glacier until we have found the furthest inland limit. Our scheme samples wider glaciers with more flowlines and finds the furthest inland thinning diffusion limit for a given glacier. We start with six flowlines, evenly spaced across glacier flow and find the thinning diffusion limit as the first location where Pe exceeds 3 upglacier of the terminus along each. Then, starting at the farthest inland thinning diffusion limit, we create six additional flowlines within the bounds of the flowlines adjacent to the one with the furthest inland thinning diffusion limit. We find thinning diffusion limits along the new set of six flowlines and identify the furthest inland. We perform three iterations this way but stop if we can not find a thinning diffusion limit further upglacier of the previous iteration or if adjacent flowlines are $<$2.5 km apart.

Text S2. Dynamic glacier thickness change

We measured dynamic thickness change by finding the total thickness change from digital elevation model (DEM) differencing and then removing the surface mass balance (SMB) component using a climate model (Fig. S1). We neglect glacial isostatic adjustment (GIA) in our measured thickness change because the largest observed uplift rates are 27.2 mm/yr (Wake et al., 2016), yielding a 1 meter error on our thickness change estimates over the observational timespan, which is less than combined DEM error (Korsgaard et

al., 2016). To obtain total thickness change, we differenced a DEM of the ice sheet surface from an aerial photo survey of Greenland ice sheet margin, collected between 1978 and 1987 (Korsgaard et al., 2016), from a DEM of the present-day ice sheet surface elevation, as measured by the Polar Geospatial Center ArcticDEM. Both DEMs are referenced to the WGS84 ellipsoid and we do not do any additional vertical or horizontal corrections before calculating the difference.

To remove the SMB component, we use output from the regional climate model, RACMO2.3p2, which has been downscaled to a spatial resolution of 1 km (Noël et al., 2019). We find the mean SMB component from 1971 to 1988, assumed to represent a time period when the ice sheet was in steady state (Rignot et al., 2008). We calculate cumulative SMB from 1985 to 2015 and calculate the SMB anomaly as the difference between the cumulative and mean SMB fields:

$$s_a = \sum_{y=1985}^{2015} s_y - \bar{s}\Delta t \tag{1}$$

where s_a represents the SMB anomaly, s_y represents the annual SMB for each year from 1985 to 2015, $\bar{s}$ represents the mean annual SMB from 1971 to 1988, and Δt represents the number of years from 1985 to 2015.

We convert SMB anomaly to glacier thickness change using a spatially-constant ice density of 917 kg / m^3 and subtract the SMB anomaly component from total thickness change from DEM differencing to obtain the dynamic thickness change. We sample dynamic thickness change along glacier centerlines and, for glaciers that have dynamically thinned, we calculate percent unit volume loss as cumulative thinning, from the terminus

to each location, as a percentage of the total cumulative thinning measured along the flowline.

Text S3. Péclet number

The behavior of terminus-initiated thinning along a glacier's flow can be modeled as a diffusive, kinematic wave (Nye, 1960). The Péclet number (Pe) is the ratio of the rate of advection to the rate of diffusion of the thinning (Cuffey & Paterson, 2010). For terminus perturbations, Pe can be used to identify locations along glacier flow upglacier of which thinning will be limited (Felikson et al., 2017). These critical locations (herein called "thinning limits") can be used to determine the susceptibility of a glacier catchment to future thinning, should thinning be initiated as the result of glacier terminus retreat.

We calculate Pe along each glacier flowline (centerlines and across-flowlines) using the ice sheet surface elevation from the aerial photo survey DEM and bed elevation from the BedMachine, v3, product. Beyond the spatial extent of the aerial photo survey DEM, we use the GIMP DEM surface topography. Bed elevations in BedMachine are referenced to the EIGEN-6C4 geoid; prior to calculating the Péclet number, we shift the vertical datum to the WGS84 ellipsoid by adding the geoid height above the ellipsoid to bed elevations. We assume that these datasets represent the geometry of the ice sheet at steady-state. To calculate Pe, we assume that ice flux is completely due to sliding and use a hard-bed sliding law (Weertman, 1957) to derive the coefficients in the kinematic wave equation used to calculate Pe. (Felikson et al., 2017) includes a complete derivation of the kinematic wave equation and justification for using the hard-bed sliding law. Because the hard-bed sliding law neglects membrane stresses, we smooth the bed and surface topography

within windows of ten times the local ice thickness, a typical longitudinal coupling length for fast-flowing ice, prior to calculating Pe (Kamb & Echelmeyer, 1986). The smoothing is done by fitting second-order polynomials to the surface topography and thickness within a window of ten times the local ice thickness at 50-meter increments along each flowline.

The Péclet number is calculated as:

$$Pe = \frac{(C_0 - \partial D_0/\partial x)}{D_0} l \tag{2}$$

where the coefficients are defined as:

$$C_0 = \left.\frac{\partial q}{\partial H}\right|_0 = K_b(m+1)H_0^m \alpha_0^m \tag{3}$$

$$D_0 = \left.\frac{\partial q}{\partial \alpha}\right|_0 = K_b(m)H_0^{m+1}\alpha_0^{m-1} \tag{4}$$

$$\frac{\partial D_0}{\partial x} = K_b m \left((m+1)H_0^m \frac{\partial H_0}{\partial x}\alpha_0^{m-1} + H_0^{m+1}(m-1)\alpha_0^{m-2}\frac{\partial \alpha_0}{\partial x} \right) \tag{5}$$

where q is ice flux, α is surface slope, H is ice thickness, m is a positive exponent in the hard-bed sliding law, and K_b is a function of the bed roughness (or slipperiness). K_b and is independent of ice thickness, H, and surface slope, α. Note that the coefficient K_b drops out of Pe equation. In this study, we use the value of $m = 1$ in the hard-bed sliding law. The coefficients of the smoothing polynomial fits represent the zeroth-, first-, and second-order derivatives of the smoothed geometry. For example, H_0 is the zeroth-order coefficient of the polynomial fit to thickness and $\partial \alpha_0/\partial x$ is the second-order coefficient of the polynomial fit to surface topography. As shown in (Felikson et al., 2017), our results are insensitive to the chosen value of m and for different formulations of the sliding law. This is because we empirically derive the value of Pe that limits thinning from diffusing

upstream. So Pe values along glacier flowlines will change with different formulations and our empirical calibration of a thinning limit will compensate.

We illustrate the Péclet number calculation along one flowline of Køge Bugt S (Fig. S2). Along this flowline, most of the observed dynamic thinning is constrained to within 6 km from the glacier's terminus (Fig. S2b). Pe remains low (<3) in this part of the glacier's flow and has a local maximum of ∼4 just upstream of the location where the bed steeply rises above sea level (Fig. S2c). Following the method of Felikson et al. (2017), we calculate the total unit volume loss along this flowline as the total cumulative thinning from the glacier's terminus along this flowline; and the "percent unit volume loss" ss the cumulative thinning from the terminus to each location along flow as a percentage of the total unit volume loss (shown as a colored curve on Fig. S2b).

For ice flow that is dominated by internal deformation, the Péclet number takes on a similar form as for hard-bed sliding because the form of the flux relationship with respect to ice thickness and surface slope. This can be readily seen by deriving the C_0, D_0, and $\partial D_0/\partial x$ coefficients using the depth-integrated, shallow-ice approximation for ice flux (Hutter, 1983):

$$q = K_b H^{n+2} \alpha^n \tag{6}$$

Taking the partial derivatives yields:

$$C_0 = \left.\frac{\partial q}{\partial H}\right|_0 = K_b(n+2)H_0^{n+1}\alpha_0^n \tag{7}$$

$$D_0 = \left.\frac{\partial q}{\partial \alpha}\right|_0 = K_b(n)H_0^{n+2}\alpha_0^{n-1} \tag{8}$$

$$\frac{\partial D_0}{\partial x} = K_b n \left((n+2)H_0^{n+1}\frac{\partial H_0}{\partial x}\alpha_0^{n-1} + H_0^{n+2}(n-1)\alpha_0^{n-2}\frac{\partial \alpha_0}{\partial x} \right) \tag{9}$$

Using the coefficients derived for hard-bed sliding (Eqns. 3, 4, and 5) and the coefficients derived for the shallow-ice approximation (Eqns. 7, 8, and 9) into the Péclet number equation (Eqn. 2):

$$Pe_{HB} = \frac{(m+1)\alpha_0}{mH_0} - \frac{(m+1)\partial H_0/\partial x}{H_0} - \frac{(m-1)\partial \alpha_0/\partial x}{m\alpha_0} \tag{10}$$

$$Pe_{SIA} = \frac{(n+2)\alpha_0}{nH_0} - \frac{(n+2)\partial H_0/\partial x}{H_0} - \frac{(n-1)\partial \alpha_0/\partial x}{n\alpha_0} \tag{11}$$

where Pe_{HB} is the Péclet number derived for hard-bed sliding and Pe_{SIA} is the Péclet number derived for the shallow-ice approximation. Although the magnitude of the terms differ, the form of both formulations is similar indicating that the magnitude of the Péclet number will differ along flow, depending on the formulation of the ice flux relationship but the along-flow pattern will remain the same. Both forms of the Péclet number are high when the glacier ice is relatively thin and the surface slopes are relatively high.

Text S4. Flowline thinning limit calibration

Calibration of an empirical threshold for inland thinning limits was done by comparing percent unit volume loss with Pe along glacier centerlines, following the method of (Felikson et al., 2017). We compare Pe with observed volume loss for only the glaciers that have been observed to have dynamically thinned between the DEM acquisition times (93 glaciers of the total 141, or 66%). We find that 89% of unit volume loss occurs downglacier of where Pe first exceeds 3 along centerlines (Fig. S3).

Text S5. Glacier-wide thinning limits

Along each flowline (centerlines and across-flowlines), we identify the location where Pe first exceeds 3 upglacier of the terminus. This location represents the "thinning limit" along each flowline; for terminus perturbations, we expect the majority of volume loss to

occur downglacier of this location. To find glacier-wide thinning limits, we collect statistics on thinning limits for each available glacier flowline (original and iterated across-flowlines). We define the "glacier-wide thinning limit" as the maximum distance from the terminus to all flowline thinning limits minus the standard deviation of those distances. For glaciers with multiple tributaries, we combined thinning limits for all flowlines from all tributaries before calculating statistics.

Text S6. Classification of regional bed topography

To separate Greenland drainage systems (Zwally et al., 2012) into ones with high relief ("mountainous") and ones with low relief ("gentle"), we use the standard deviation of bed elevations within each drainage system (Table S1). Basins in which the standard deviation of bed elevations is larger than 300 m are labeled as mountainous; all others are gentle.

Text S7. Knickpoint characterization

To identify knickpoints, we use a procedure that searches for local minima and maxima in the moving-averaged bed, sampled along flowlines. We use the Matlab function *findpeaks* to identify all local minima and maxima that exceed a prominence of 5 m. We found that this threshold balances identifying the prominent local minima and maxima while minimizing identification of local minima and maxima that are present due to small errors in the bed topography. We then calculate the vertical rise of the bed between each local minimum and the subsequent local maximum and label the rises greater than 300 m as "large rises." We combine large rises that are proximal to one another if the local minimum of one rise is within 10 km horizontally and 100 m vertically of the previous local

maximum (Fig. S4). To further characterize the bed, we perform linear regression to fit lines through bed elevations within three regions: the large rise at sea level, downstream, and upstream of the large rise at sea level.

We define a knickpoint using four criteria:

1. Our algorithm has found a large rise in the bed (>300 m) at the location where the bed first crosses sea-level upstream of the terminus.

2. The large rise at sea level represents a step change in bed elevation such that the mean elevation plus one standard deviation within the trough is lower than the mean minus one standard deviation upstream of the large rise.

3. The slope from linear regression of the large rise at sea level is higher than the slope downstream of the large rise.

4. The extent of the large rise at sea level occurs over a relatively short distance (<100 km).

Along some flowlines, our algorithm cannot identify whether or not a knickpoint exists. This occurs when a local minimum cannot be identified downstream of the location where the bed crosses sea-level.

As one example, we show the results of the algorithm along a flowline at Helheimgletscher (Fig. S4). A knickpoint is identified here as the large step in the bed at sea level.

Text S8. Glacier catchment ice flux

To measure glacier catchment ice flux, we sample surface speed and ice thickness within 5 km of each glacier's terminus. Surface speed was sampled from the MEaSUREs Annual

Greenland Outlet Glacier Terminus Positions from SAR Mosaics, Version 1 dataset (Moon & Joughin, 2008; I. Joughin et al., 2015, 2017) and ice thickness was obtained from the Greenland Ice Mapping Project (Howat et al., 2014) and the BedMachine product (Morlighem et al., 2017; Morlighem, 2017). Glacier width was estimated as the distance between the furthest across-flowlines along a line perpendicular to the centerline within the 5 km window. Ice flux for each glacier was estimated as the maximum ice flux found within this window.

Text S9. Uncertainty in input datasets

We conservatively assume that the input datasets have systematic (spatially correlated) errors and determine how these offsets affect (1) calibration of the Pe=3 thinning limit and (2) locations of the glacier-wide thinning limits. We quantify dataset uncertainties along each glacier flow line and propagate them through our entire analysis.

Examining discrepancies between BedMachine and radar-derived bed topography along glacier flow lines in West Greenland shows that BedMachine is able to reproduce the long-wavelength shape of the bed but systematically underestimates the thickness of the ice by 10% - 20% (Figs. S1-S7 in Catania et al. (2018)). Based on these comparisons, we conservatively assume that along each glacier flow line, the bed can be deeper by up to 20% of ice thickness:

$$\sigma_b = 0.20 \times H \tag{12}$$

where H is the ice thickness along each flow line and σ_b is the systematic uncertainty in the bed.

The aerial photo DEM was created from stereo imagery collected between 1978 and 1987 (Korsgaard et al., 2016); for brevity, we refer to this data product as the "1985 DEM." In West Greenland, errors in the 1985 DEM elevations vary from ∼5 m along the ice sheet margin, where sufficient ground control exists, to ∼20 m in the interior (Fig. S5) (Felikson et al., 2017). We assume that the structure of the uncertainty in the aerial photo DEM around the entire ice sheet is the same as the structure in West Greenland. Thus, we conservatively assume that error in the aerial photo DEM varies from 5-20 m (within the spatial extent of the aerial photo DEM) along each glacier flow line around the GrIS.

$$\sigma_{h_{1985}} = 5 + \frac{15}{l}x \tag{13}$$

where x is the coordinate along the flowline and l is the total distance from the terminus to the last valid aerial photo DEM elevation along the flowline.

The ArcticDEM, release 6, is constructed as a mosaic of DEM strips collected by the DigitalGlobe constellation between 2008 and 2017 (Porter et al., 2018). For brevity, we refer to this data product as the "2014 DEM." DEM strips are accurate to within 0.2 m, validated by NASA Airborne Topographic Mapper (ATM) LIDAR (Noh & Howat, 2015). Information is not provided on planar slope errors or other systematic biases. Therefore, we assume that the ArcticDEM has 0.2 m of random error and no systematic offsets. Prior to calculating % unit volume loss, we smooth topography over 10 ice thicknesses, and essentially average out all random errors in the ArcticDEM. Thus, we neglect error in the ArcticDEM for the remainder of the analysis.

The GIMP DEM is a data product that represents an average ice sheet surface elevation between 2003 and 2009. To represent error in the GIMP DEM elevation measurements, we use a conservative 10% of the elevation in each grid cell. This offset is used to systematically shift the elevation measurement high and low in the same manner as for the aerial photo DEM.

RACMO surface mass balance is the difference between accumulation and ablation:

$$s = acc - abl \tag{14}$$

Accumulation is the sum of precipitation and water vapor deposition and we assume that water vapor deposition can be neglected. Ablation is the sum of runoff and sublimation and we assume that sublimation can be neglected. Thus, SMB can be approximated as the difference between precipitation and runoff:

$$s = p - r \tag{15}$$

Noël et al. (2019) use 213 stake measurements from the GrIS ablation zone and 182 stake measurements from the accumulation zone to evaluate the RACMO2.3p2 SMB output. Van As et al. (2018) and Noël et al. (2019) use measurements of discharge from the Watson River, which is fed almost entirely by ice sheet melt, to evaluate RACMO2.3p2 runoff over a catchment. These papers find 10% error in RACMO2.3p2 accumulation and 20% in runoff and we assume the error in runoff is equal to the error in ablation. We propagate these as systematic errors through our calculation of the SMB anomaly. Because RACMO2.3p2 SMB is provided in units of mass, we convert to units of height by assigning uncertainties to the densities of the surface in the ablation and accumulation zones, separately. The calculation is detailed below.

Assuming that accumulation is equal to precipitation ($a = p$), we combine uncertainties from accumulation (10%) and ablation (20%) in quadrature to obtain uncertainty in SMB:

$$\sigma_s^2 = (0.1 \times a)^2 + (0.2 \times r)^2 \tag{16}$$

where σ_s represents the uncertainty in the annual SMB, a represents annual accumulation, and r represents annual runoff within a given grid cell.

Conservatively, we assume that uncertainty is spatially correlated such that σ_s represents an offset, positive or negative, in SMB along an entire flow line. But, we assume that from one year to the next σ_s is uncorrelated and SMB uncertainties from one year to the next are independent. To obtain the uncertainty on SMB anomaly, defined as the difference between cumulative SMB from 1985-2014 and SMB from a period of time when the ice sheet was in balance (Rignot et al., 2008), accumulated over the time span over which we measure thinning using the DEMs (1985-2014), we calculate:

$$\sigma_{s_a}^2 = \sum_{i=1985}^{2014} \sigma_{s_i}^2 + \left(\frac{n}{m}\right)^2 \sum_{i=1979}^{1988} \sigma_{s_i}^2 \tag{17}$$

where σ_{s_a} is the uncertainty in the 1985-2014 SMB anomaly in units of mass, n is the number of years between 1985 and 2014 (the timespan over which dynamic thinning is measured), and m is the number of years between 1979 and 1988 (the timespan over which the SMB anomaly is referenced).

Additionally, we account for uncertainty in the conversion from units of mass to height by assigning uncertainties to the densities used in the conversion. In the ablation zone, we assume that the surface density is that of bare ice with some uncertainty and we assign the density to be 800 ± 100 kg/m^3, or 800 kg/m^3 $\pm$ 12.5%. In the accumulation zone,

we assume that the surface density can be between that of fresh snow and bare ice and we assign the density to be 600 ± 300 kg/m^3, or 600 kg/m^3 $\pm$ 50%. The ablation zone is defined to be where $\bar{s} \leq 0$ and the accumulation zone is defined to be where $\bar{s} > 0$. We add the density error as a percentage to σ_{s_a} to calculate the error in units of height:

$$\sigma_{h_{s_a}} = \begin{cases} \sigma_{s_a} \times 1.125, & \bar{s} \leq 0 \\ \sigma_{s_a} \times 1.5, & \bar{s} > 0 \end{cases} \tag{18}$$

This gives us a spatially varying structure in uncertainties on the 1985-2014 SMB anomaly in units of height (Fig. S6).

Text S10. Uncertainty in Peclet number

We use the dataset uncertainties to quantify systematic uncertainties in our calculation of dynamic thinning and the Péclet number. Systematic uncertainties in the input datasets propagate through our calculations of the two derived quantities by biasing each quantity low or high, as described below. These describe the "worst-case" upper and lower bounds for the two derived quantities.

Dynamic thinning is calculated as:

$$\Delta h = h_{2014} - h_{1985} - h_{s_a} \tag{19}$$

Therefore, systematic uncertainties in the ArcticDEM, the aerial photo DEM, and SMB anomaly can combine to bias the dynamic thinning calculation low or high. We assume that there is no systematic error in the ArcticDEM, so the upper and lower bounds on dynamic thinning are:

$$\Delta h_{high} = \Delta h - \sigma_{h_{1985}} - \sigma_{s_a} \tag{20}$$

$$\Delta h_{low} = \Delta h + \sigma_{h_{1985}} + \sigma_{s_a} \tag{21}$$

where $\sigma_{h_{1985}}$ is calculated using Eqn. 13 and σ_{s_a} is calculated using Eqn. 18.

Similarly to dynamic thinning, the Péclet number (Pe) can be systematically biased depending on the combination of uncertainties in the input datasets. We calculate Pe using the combinations of systematically offset bed and surface topography that produce the largest range of calculated Pe:

$$Pe_{low}^{high} = Pe(h_{1985} + \sigma_{h_{1985}}, b - \sigma_b) \tag{22}$$

where Pe is calculated with a "high" 1985 DEM and "low" bed.

$$Pe_{low}^{low} = Pe(h_{1985} - \sigma_{h_{1985}}, b - \sigma_b) \tag{23}$$

where Pe is calculated with a "low" 1985 DEM and "low" bed.

We illustrate our approach along two flow lines (Fig. S7). The top panels show the nominal bed geometry (black) and shifted low (red) using the systematic error described by Eqn. 12. The 1985 DEM surface is shown shifted high (green) and low (blue), calculated by adding and subtracting the systematic error given by Eqn. 13. The middle panels show the original calculated dynamic thinning (black), dynamic thinning biased high (green), and dynamic thinning biased low (blue). The bottom panels show the original Pe (black), Pe biased high (green), and Pe biased low (blue).

Text S11. Uncertainty in Pe=3 thinning limit calibration

To analyze the effect of dataset uncertainties on this empirical calibration, we use two approaches. In the first approach, we randomly choose Pe and dynamic thinning along each center line calculated from either (1) a high 1985 DEM and low bed or (2) a low 1985 DEM and low bed. For each randomly drawn realization of thinning glaciers, we find the median % unit volume loss for each Péclet number (Felikson et al., 2017). We

create 100 realizations of biased dynamic thinning and Pe and quantify statistics across all realizations. For some glaciers, by biasing the dynamic thinning low, we can no longer measure the complete spatial extent of dynamic thinning within the coverage of the 1985 DEM. Because of this, in each realization, we keep only those center lines along which the furthest inland dynamic thinning measurement is $\leq$15% of the dynamic thinning at the terminus. Pseudo-code that describes the algorithm is below and results are shown in Fig. S8.

Pseudo-code – approach 1

```
for i = 1 to 100:  # 100 realizations
  for g = 1 to 93: # 93 thinning glaciers (extent of thinning within DEM)
    # Randomly select a combination
    choice = random_integer([1,2])
    if choice == 1:
      Pe = glacier[g]["Pe_hilo"] # Pe from high DEM / low bed (Eqn. 20)
      dh = glacier[g]["dh_hi"]   # dynamic dh from high DEM (Eqn. 18)
    if choice == 2:
      Pe = glacier[g]["Pe_lolo"] # Pe from low DEM / low bed (Eqn. 21)
      dh = glacier[g]["dh_lo"]   # dynamic dh from low DEM (Eqn. 19)

    # Decide whether to include this glacier
    if abs(dh[-1]) < 0.15*abs(dh[0]):

      Pe_runmax = runningMax(Pe) # Pe running maximum
      unitvolloss = unitvolloss(dh) # unit volume loss

      # Find glacier medians within windows of width Pe=1
      Pe_med[g], unitvolloss_med[g] = runningMedian(Pe_runmax, unitvolloss, window=1)

  # Find medians of glacier medians
  Pe_medmed[i], unitvolloss_medmed[i] = runningMedian(Pe_med, unitvolloss_med, window=1)

  # Black boxplots in Fig. S8 represent statistics on Pe_medmed
```

In the second approach, we assume that there is equal chance of drawing derived quantities from the following combinations of input datasets: (1) nominal 1985 DEM surface and nominal bed, (2) high 1985 DEM surface and low bed, and (3) low 1985 DEM surface and low bed. Thus, we use each of the three combinations of derived quantities along each center line. Similar to the first approach, we only select those glaciers that satisfy our inclusion criteria for all three dynamic thinning realizations. Results are shown in Fig. S9. After accounting for the propagation of systematic errors in the input datasets

through our calculation of Pe, we look for the value of Pe that limits upstream diffusion of thinning. To do this, we identify the lowest Pe value at which >75% of unit volume loss occurs downstream. In other words, <25% of unit volume loss occurs upstream of the location where Pe first exceeds this value. With Approach 1, we find that in 100 realizations of randomly sampled systematically offset measurements of Pe and dynamic dh, the first quartile of median unit volume loss is >75% at Pe=2 (black whisker plots in Fig. S8). With Approach 2, we find that the first quartile of median unit volume loss >75% at Pe=3 (black whisker plots in Fig. S9). Thus, we argue that Pe=2 and Pe=3 are both appropriate measures of the location along glacier flow beyond which we expect limited dynamic thinning diffusing from terminus perturbations. We include uncertainty in the choice of a particular Pe value as the empirical thinning limit by using Pe=2, Pe=3, and Pe=4 as empirical thinning limits in our Monte Carlo error sampling.

Pseudo-code – approach 2

```
for g = 1 to 93: # 93 thinning glaciers
  # Decide whether to include this glacier
  if include1 and include2 and include3:
    Pe_nom = glacier[g]["Pe_nominal"] # Pe from nominal DEM / nominal bed
    dh_nom = glacier[g]["dh_nominal"]   # dynamic dh from nominal DEM
    Pe_hi = glacier[g]["Pe_hilo"] # Pe from high DEM / low bed (Eqn. 20)
    dh_hi = glacier[g]["dh_hi"]   # dynamic dh from high DEM (Eqn. 18)
    Pe_lo = glacier[g]["Pe_lolo"] # Pe from low DEM / low bed (Eqn. 21)
    dh_lo = glacier[g]["dh_lo"]   # dynamic dh from low DEM (Eqn. 19)

    if abs(dh_nom[-1]) < 0.15*abs(dh_nom[0]) and
       abs(dh_hi[-1])  < 0.15*abs(dh_hi[0]) and
       abs(dh_lo[-1])  < 0.15*abs(dh_lo[0]):

       Pe_runmax_nom = runningMax(Pe_nom) # Pe running maximum
       unitvolloss_nom = unitvolloss(dh_nom) # % unit volume loss
       Pe_runmax_hi = runningMax(Pe_hi) # Pe running maximum
       unitvolloss_hi = unitvolloss(dh_hi) # % unit volume loss
       Pe_runmax_lo = runningMax(Pe_lo) # Pe running maximum
       unitvolloss_lo = unitvolloss(dh_lo) # % unit volume loss

       # Find glacier medians within windows of width Pe=1
       Pe_med_nom, unitvolloss_med_nom = runningMedian(Pe_runmax_nom, unitvolloss_nom)
       Pe_med_hi, unitvolloss_med_hi = runningMedian(Pe_runmax_hi, unitvolloss_hi)
       Pe_med_lo, unitvolloss_med_lo = runningMedian(Pe_runmax_lo, unitvolloss_lo)

       # Append
       Pe_med_all.append(Pe_med_nom)
       Pe_med_all.append(Pe_med_hi)
```

```
        Pe_med_all.append(Pe_med_lo)
        unitvolloss_med_all.append(unitvolloss_med_nom)
        unitvolloss_med_all.append(unitvolloss_med_hi)
        unitvolloss_med_all.append(unitvolloss_med_lo)

# Find medians of glacier medians
Pe_medmed, unitvolloss_medmed = runningMedian(Pe_med_all, unitvolloss_med_all, window=1)

# Black boxplots in Fig. S9 represent statistics on Pe_medmed
```

Text S12. Uncertainty in glacier-wide thinning limits

To create one metric that can be used to evaluate the distance to which thinning can spread along each glacier, we combine all Pe empirical thinning limits from all across-flowlines; we call this the glacier-wide thinning limit. As shown in the previous section, there is uncertainty in the location of the thinning limit along each across-flowline because of (1) uncertainty in the calculation of the Péclet number due to uncertainties in input datasets and (2) uncertainty in our selection of Pe=3 as the empirical thinning limit. Here, we investigate how our calculation of glacier-wide thinning limits is impacted as the result of these sources of uncertainty. To do this, we create 100 realizations of randomly sampled biased Pe along each across-flowline for each glacier. For each realization, we calculate the glacier-wide thinning limit.

Pseudo-code – glacier-wide thinning limit error

```
for g = 1 to 141:
  for PeThreshold = 2 to 4:
    for i = 1 to 100:
      for a = 1 to n_across_flowlines: # including iterated flowlines
        choice = random.randint(1,3)
        if choice == 1:
          Pe = glacier[g][a]["Pe_nominal"]
        if choice == 2:
          Pe = glacier[g][a]["Pe_hilo"]
        if choice == 3:
          Pe = glacier[g][a]["Pe_lolo"]

        Pe_thresh_distances[a] = findThinningLimit(Pe, PeThreshold=PeThreshold)

      glacier_wide_thinning_limits[g][i] = glacierWideThinningLimit(Pe_thresh_distances)
```

The ranges of glacier-wide thinning limits for each glacier are shown in Fig. S10. For glaciers in mountainous topography, the minimum and maximum thinning extents coincide, as expected; the thinning limits for these glaciers tend to be set by steep knickpoints, where the Péclet number tends to be high. Thus, the glacier-wide thinning limits are not sensitive to uncertainty in glacier geometry nor our selection of the empirical value of Pe and thus we have a high confidence in the spatial extent of thinning for these glaciers.

For glaciers in gentle topography, there are large discrepancies between the minimum and maximum thinning extents and this is also expected; these glaciers lack steep knickpoints and glacier-wide thinning limits are more sensitive to uncertainties. Thus, we have lower confidence in the exact thinning limit for these glaciers. This strengthens our assertion that glaciers in regions of gentle bed topography are more susceptible to far inland thinning and may contribute as much to future sea-level rise as higher-flux glaciers in regions mountainous topography. Thus, the glacier-wide thinning limits we show in Fig. 3b of the main text are conservative and thinning can extend further inland in regions of gentle topography than is shown there.

References

Catania, G. A., Stearns, L. A., Sutherland, D. A., Fried, M. J., Bartholomaus, T. C., Morlighem, M., ... Nash, J. (2018, August). Geometric Controls on Tidewater Glacier Retreat in Central Western Greenland. *Journal of Geophysical Research: Earth Surface*, *29*(1), 1–15.

Cuffey, K., & Paterson, W. S. B. (2010). *The Physics of Glaciers* (4th ed.). Academic Press.

Felikson, D., Bartholomaus, T. C., Catania, G. A., Korsgaard, N. J., Kjær, K. H., Morlighem, M., ... Nash, J. D. (2017, April). Inland thinning on the Greenland ice sheet controlled by outlet glacier geometry. *Nature Geoscience*, *10*(5), 366–369.

Howat, I. M., & Eddy, A. (2011, July). Multi-decadal retreat of Greenland's marine-terminating glaciers. *Journal of Glaciology*, *57*(203), 1–8.

Howat, I. M., Negrete, A., & Smith, B. E. (2014). The Greenland Ice Mapping Project (GIMP) land classification and surface elevation data sets. *The Cryosphere*, *8*(4), 1509–1518.

Hutter, K. (1983). *Theoretical glaciology: material science of ice and the mechanics of glaciers and ice sheets.* Dordrecht, Netherlands: Reidel Publishing.

Joughin, I., Moon, T., Joughin, J., & Black, T. (2015, 2017). *MEaSUREs Annual Greenland Outlet Glacier Terminus Positions from SAR Mosaics, Version 1.* Retrieved from http://dx.doi.org/10.5067/DC0MLBOCL3EL (Boulder, Colorado USA. NASA National Snow and Ice Data Center Distributed Active Archive Center) doi: 10.5067/DC0MLBOCL3EL

Joughin, I., Smith, B., Howat, I., & Scambos, T. (2016). *MEaSUREs Multi-year Greenland Ice Sheet Velocity Mosaic, Version 1.* Retrieved from http://dx.doi.org/10.5067/QUA5Q9SVMSJG (Boulder, Colorado USA. NASA National Snow and Ice Data Center Distributed Active Archive Center) doi: 10.5067/QUA5Q9SVMSJG

Joughin, I. R., Smith, B. E., & Howat, I. M. (2017, November). A complete map of Greenland ice velocity derived from satellite data collected over 20 years . *Journal of Glaciology*, *56*.

Kamb, B., & Echelmeyer, K. A. (1986, January). Stress-gradient coupling in glacier flow: I. Longitudinal averaging of the influence of ice thickness and surface slope. *Journal of Glaciology*, *32*(111).

Korsgaard, N. J., Nuth, C., Khan, S. A., Kjeldsen, K. K., Bjørk, A. A., Schomacker, A., & Kjær, K. H. (2016, May). Digital elevation model and orthophotographs of Greenland based on aerial photographs from 1978–1987. *Scientific Data*, *3*, 160032.

Moon, T., & Joughin, I. R. (2008, June). Changes in ice front position on Greenland's outlet glaciers from 1992 to 2007. *Journal of Geophysical Research*, *113*, F02022.

Morlighem, M. (2017). *IceBridge BedMachine Greenland, Version 3.* Retrieved from http://dx.doi.org/10.5067/2CIX82HUV88Y (Boulder, Colorado USA. NASA National Snow and Ice Data Center Distributed Active Archive Center) doi: 10.5067/2CIX82HUV88Y

Morlighem, M., Williams, C. N., Rignot, E., An, L., Arndt, J. E., Bamber, J. L., ... Zinglersen, K. B. (2017, November). BedMachine v3: Complete Bed Topography and Ocean Bathymetry Mapping of Greenland From Multibeam Echo Sounding

Combined With Mass Conservation. *Geophysical Research Letters*, *44*(21), 11,051–11,061.

Murray, T., Scharrer, K., Selmes, N., Booth, A. D., James, T. D., Bevan, S. L., ... McGovern, J. (2015, August). Extensive Retreat of Greenland Tidewater Glaciers, 2000–2010. *Arctic, Antarctic, and Alpine Research*, *47*(3), 427–447.

Noël, B. P. Y., van de Berg, W. J., Lharmitte, S., & van den Broeke, M. R. (2019, September). Rapid ablation zone expansion amplifies north Greenland mass loss. *Science Advances*, *5*.

Noh, M.-J., & Howat, I. M. (2015, February). Automated stereo-photogrammetric DEM generation at high latitudes: Surface Extraction with TIN-based Search-space Minimization (SETSM) validation and demonstration over glaciated regions. *GIScience & Remote Sensing*, *52*(2), 198–217.

Nye, J. F. (1960, July). The response of glaciers and ice-sheets to seasonal and climatic changes. *Proceedings of the Royal Society of London Series A, Mathematical and Physical Sciences*, *256*, 559–584.

Porter, C., Morin, P., Howat, I., Noh, M.-J., Bates, B., Peterman, K., ... Bojesen, M. (2018). *ArcticDEM.* (Harvard Dataverse, V1) doi: 10.7910/DVN/OHHUKH

Rignot, E., Box, J. E., Burgess, E., & Hanna, E. (2008, October). Mass balance of the Greenland ice sheet from 1958 to 2007. *Geophysical Research Letters*, *35*(20), L20502.

Rignot, E., & Mouginot, J. (2012, June). Ice flow in Greenland for the International Polar Year 2008-2009. *Geophysical Research Letters*, *39*, L11501.

Van As, D., Hasholt, B., Ahlstrøm, A. P., Box, J. E., Cappelen, J., Colgan, W. T., ... van den Broeke, M. R. (2018, June). Reconstructing Greenland Ice Sheet meltwater discharge through the Watson River (1949–2017). *Arctic, Antarctic, and Alpine Research*, *50*(1), 1–11.

Wake, L. M., Lecavalier, B. S., & Bevis, M. G. (2016, July). Glacial Isostatic Adjustment (GIA) in Greenland: a Review. *Current Climate Change Reports*, *2*, 101–111.

Weertman, J. (1957, March). On the sliding of glaciers. *Journal of Glaciology*, *3*, 33–38.

Zwally, H. J., Giovinetto, M. B., Beckley, M. A., & Saba, J. L. (2012). *Antarctic and Greenland Drainage Systems, GSFC Cryospheric Sciences Laboratory.* (Data accessed at http://icesat4.gsfc.nasa.gov/cryo_data/ant_grn_drainage_systems.php)

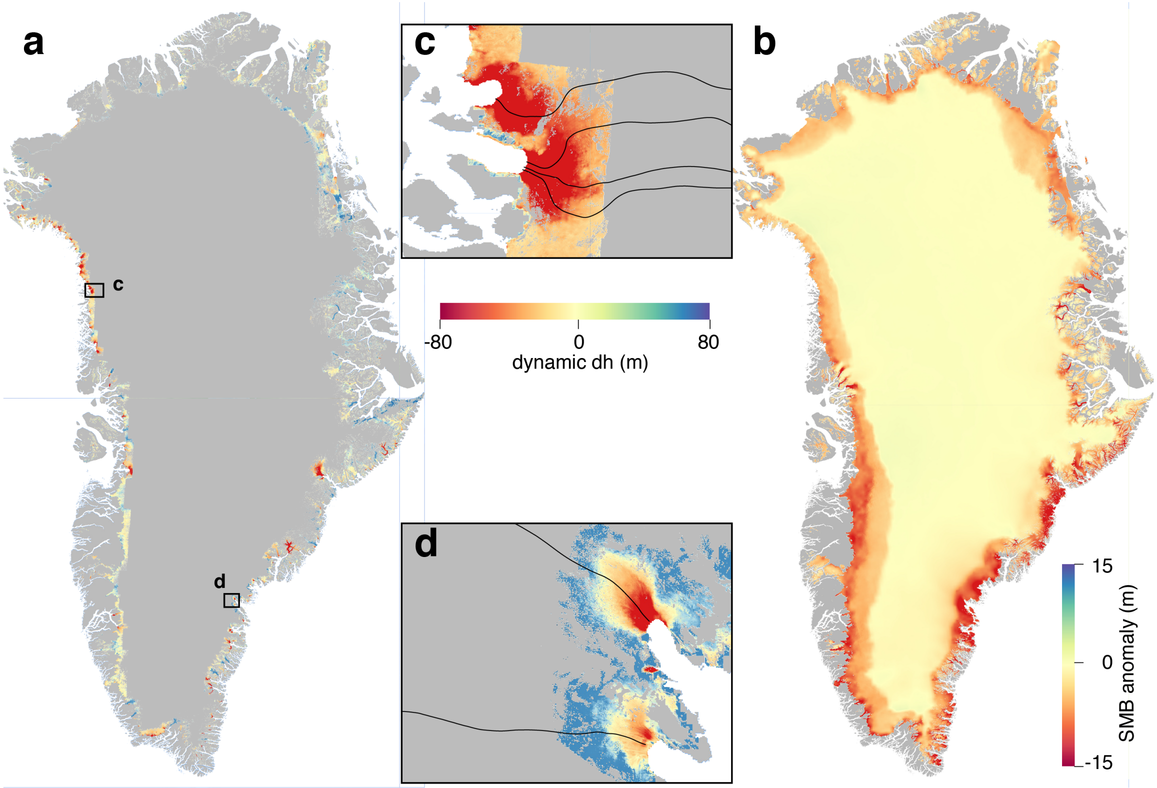

Figure S1. **(a)** Dynamic thickness change around the GrIS. **(b)** SMB anomaly from 1985-2015 (note the different colorscale for SMB anomaly). **(c,d)** Glacier centerlines showing glaciers that have dynamically thinned beyond the spatial extent of the DEM difference (c) and those for which dynamic thinning is within the spatial extent of the DEM difference (d). Data gaps in dynamic thickness change are caused by data gaps in the aerial photo DEM because of failed feature matching over steep or low-contrast terrain.

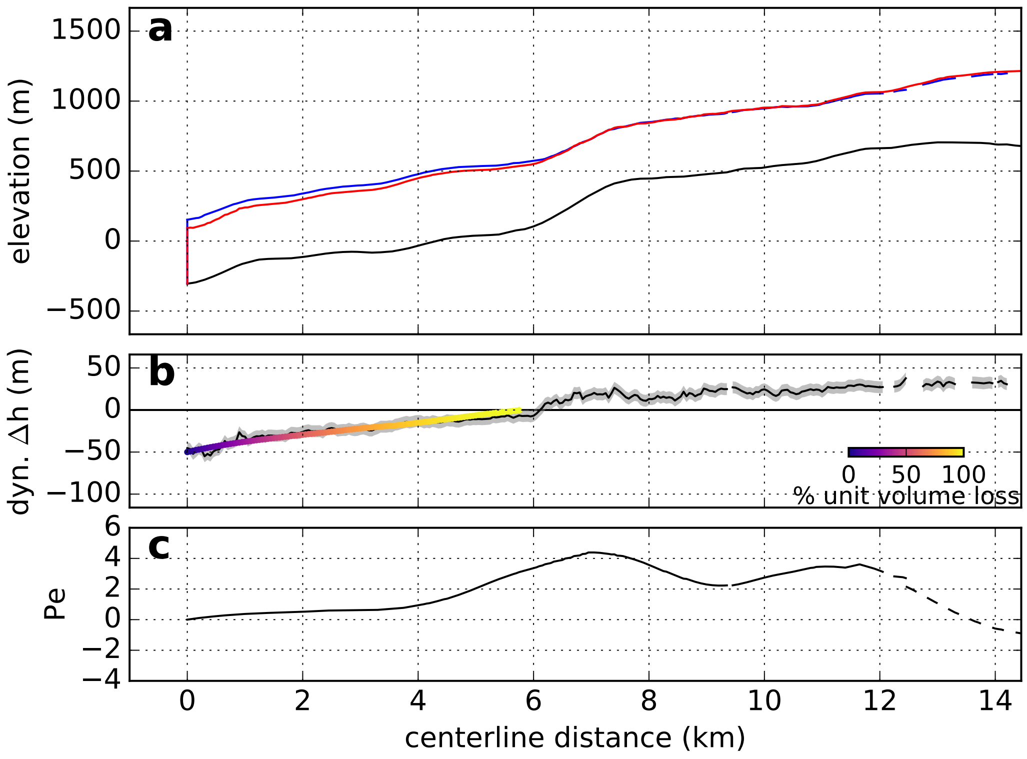

Figure S2. Geometry, thinning, and Péclet number along the flow of Køge Bugt S. **(a)** Glacier surface from aerial photo DEM (blue), glacier surface from ArcticDEM (red), and bed topography from BedMachine (black). **(b)** Dynamic thinning along flow (black) and % unit volume loss (color) along the flowline. **(c)** Péclet number along the flowline.

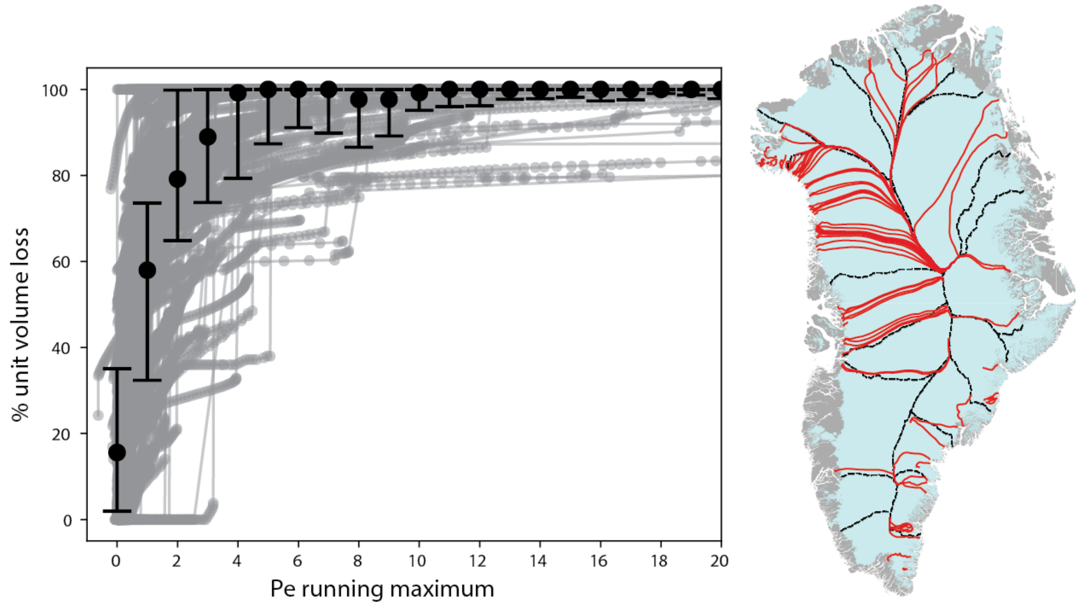

Figure S3. Volume loss and Peclet numbers along 93 TWG centerlines around the GrIS. Percent unit volume loss and running Peclet maxima for the selected glaciers are shown in the plots (left) with median values (black dots) and 25th and 75th percentiles (black bars) shown for windows of width equal to 1 Pe. Maps (right) shows glacier centerlines that were used to create the plots (red).

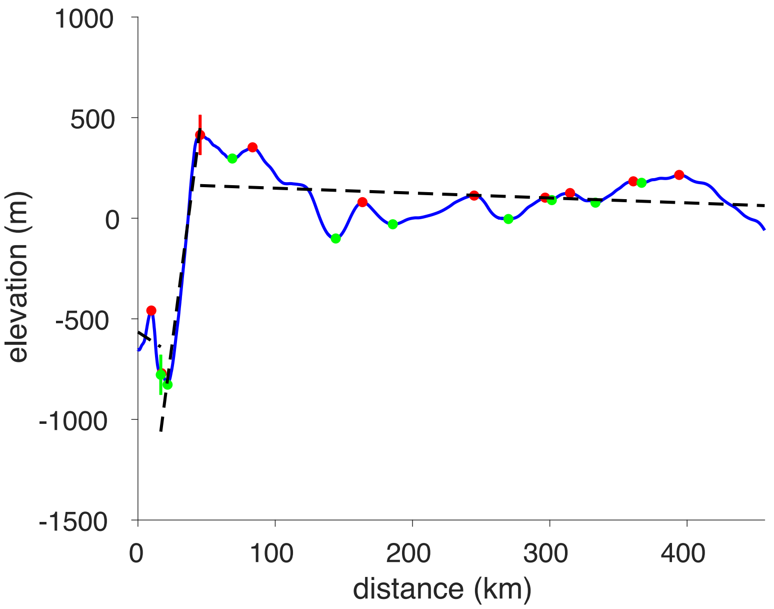

Figure S4. Example of the knickpoint identification algorithm along a Helheimgletcher flowline. All local maxima and minima shown as green and red dots, respectively. Green and red vertical lines represent start and end of large bed rises along both flowlines; thicker green and red vertical lines indicate the sea-level knickpoint. Black lines depict linear regression fits to bed elevations in the troughs, at the knickpoint, and upglacier.

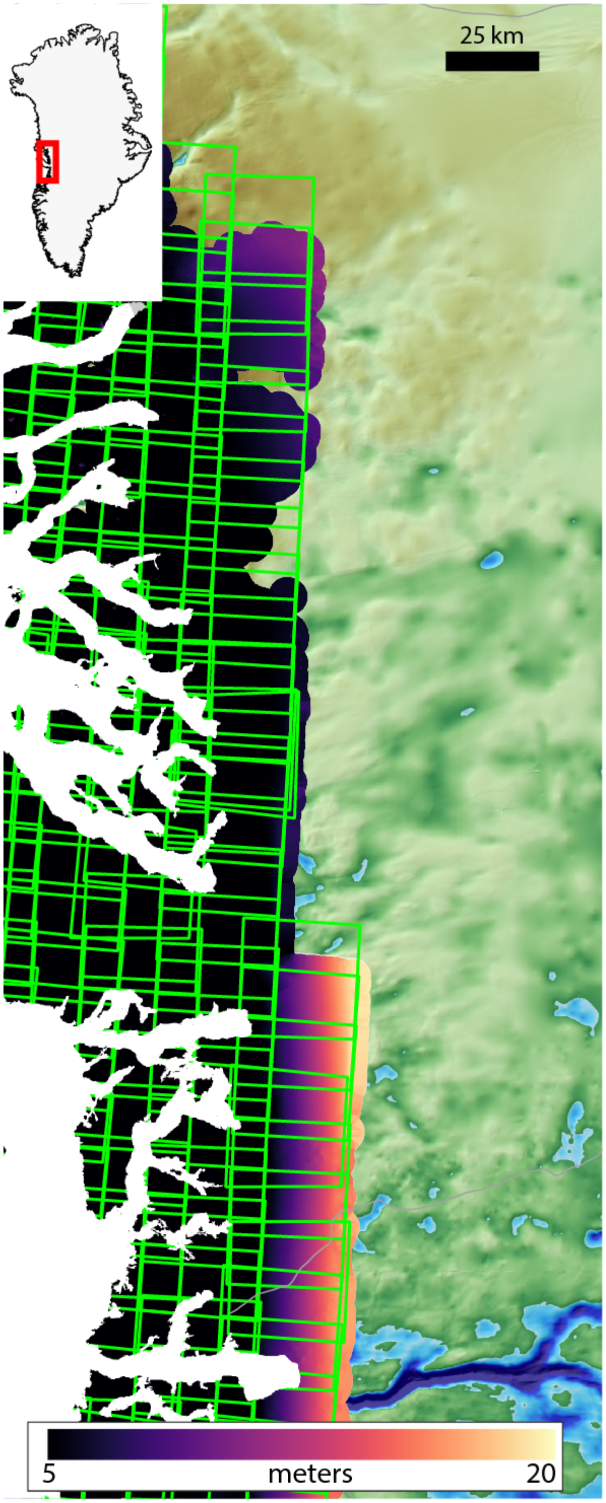

Figure S5. Spatially varying uncertainty in aerial photo DEM over West GrIS. Green outlines show footprints of individual photos. The Jakobshavn Isbræ trough can be seen at the southern end of the region.

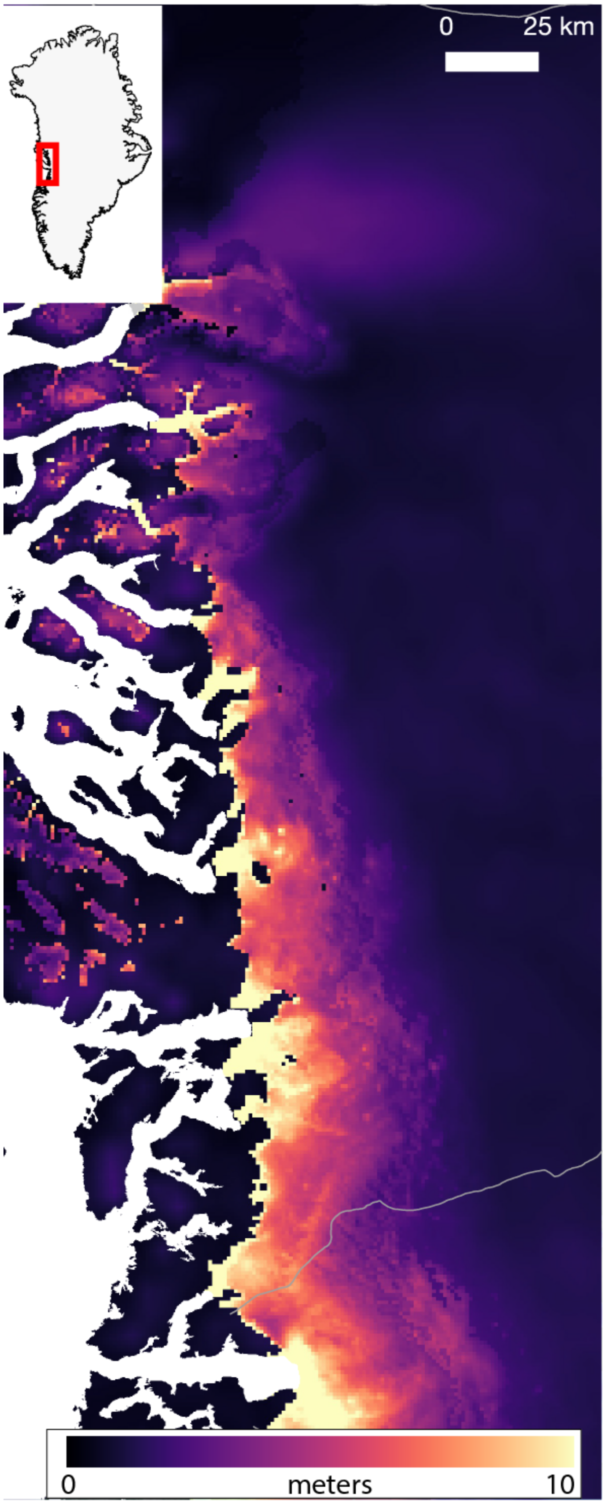

Figure S6. Uncertainty in RACMO2.p2 SMB anomaly in West Greenland.

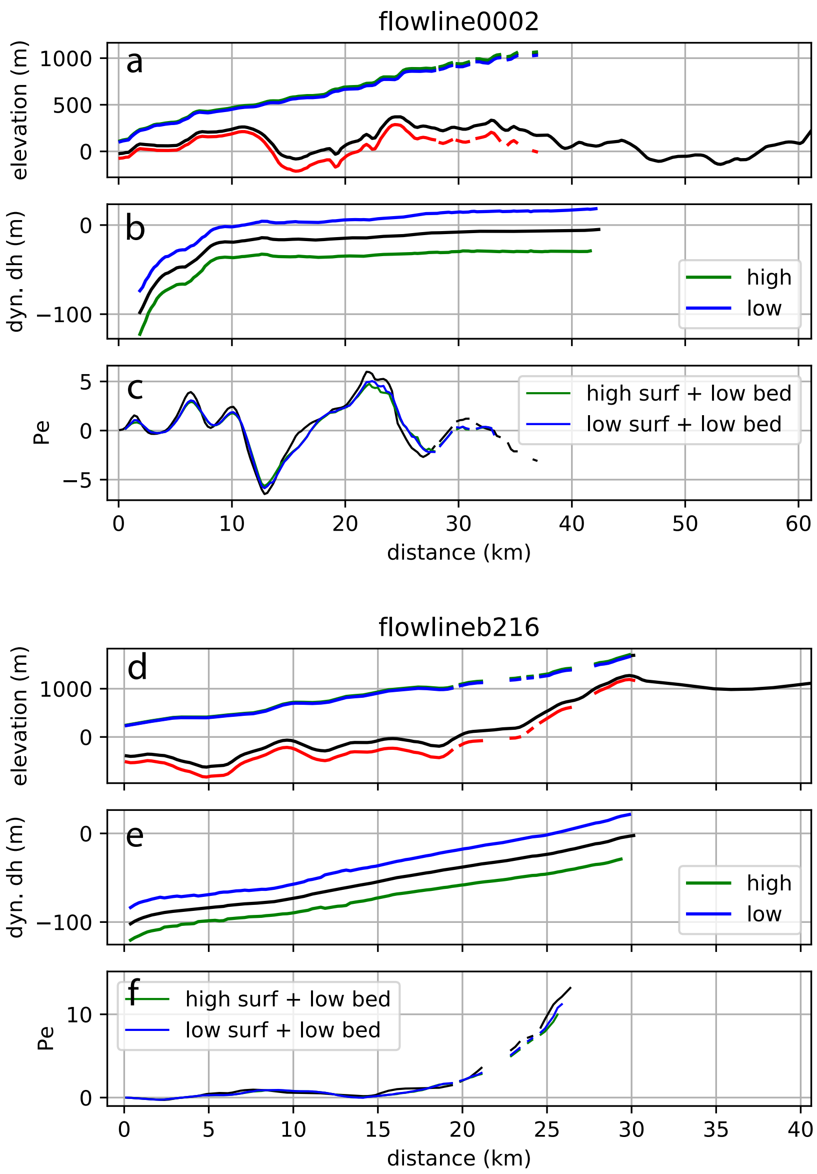

Figure S7. Examples of systematic errors along flow lines. **(a)** Glacier surface and bed topography along centerline 0002. Black color shows nominal topography, red shows offset bed topography, green and blue show high and low surface topography, respectively. **(b)** Dynamic thinning. Black shows nominal calculation, green and blue show calculation using high and low surface topography, respectively. **(c)** Peclet number. Black shows nominal calculation with Pe_{low}^{high} (green) and Pe_{low}^{low} (blue) also shown. **(d-f)** Same as **(a-c)** but for centerline b216.

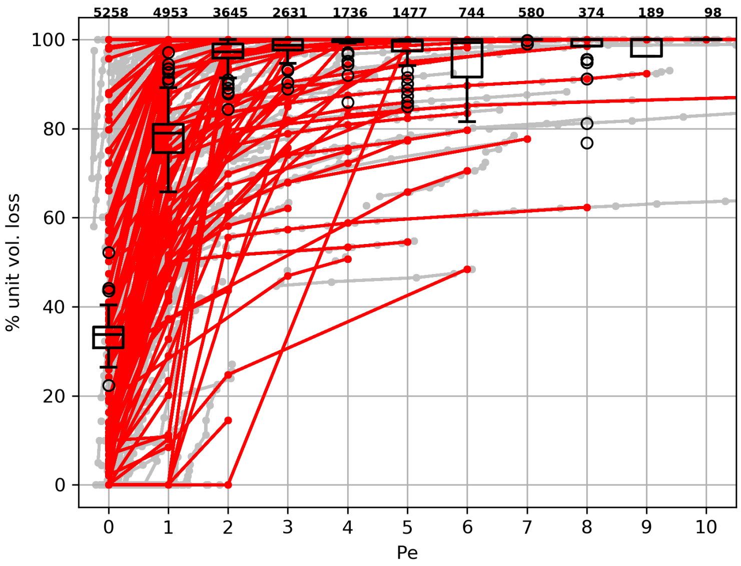

Figure S8. Volume loss and Peclet numbers for 100 realizations of errors along centerlines for thinning glaciers. Percent unit volume loss and running Peclet maxima for the glaciers are shown in gray, glacier medians in red, and medians of glacier medians as black box plots for windows of width equal to 1 Pe. The values shown along the top of the plot represent the total number of glacier medians within that Pe window from all 100 realizations.

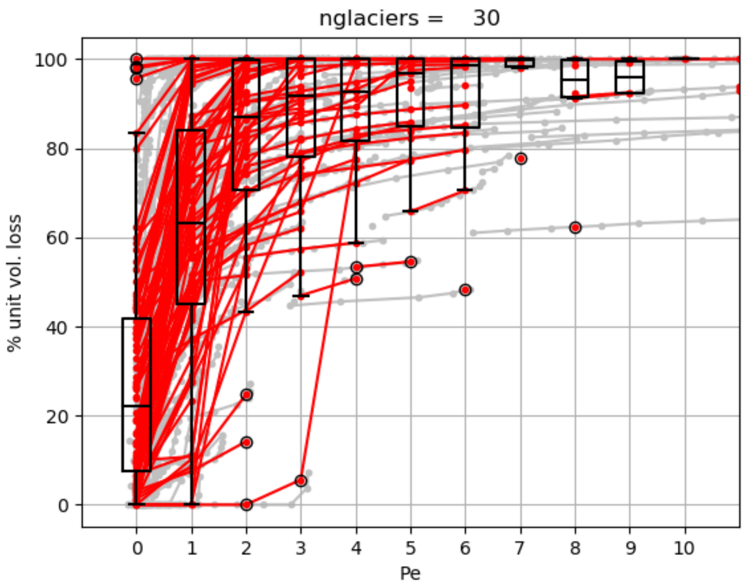

Figure S9. Volume loss and Peclet numbers for three combinations of sampled uncertainties along centerlines for thinning glaciers. Percent unit volume loss and running Peclet maxima for the glaciers are shown in gray, glacier medians in red, and medians of glacier medians as black box plots for windows of width equal to 1 Pe. Plot title indicates that 30 glaciers have been included in analysis for which extent of thinning is within coverage of DEM difference (see text for more details).

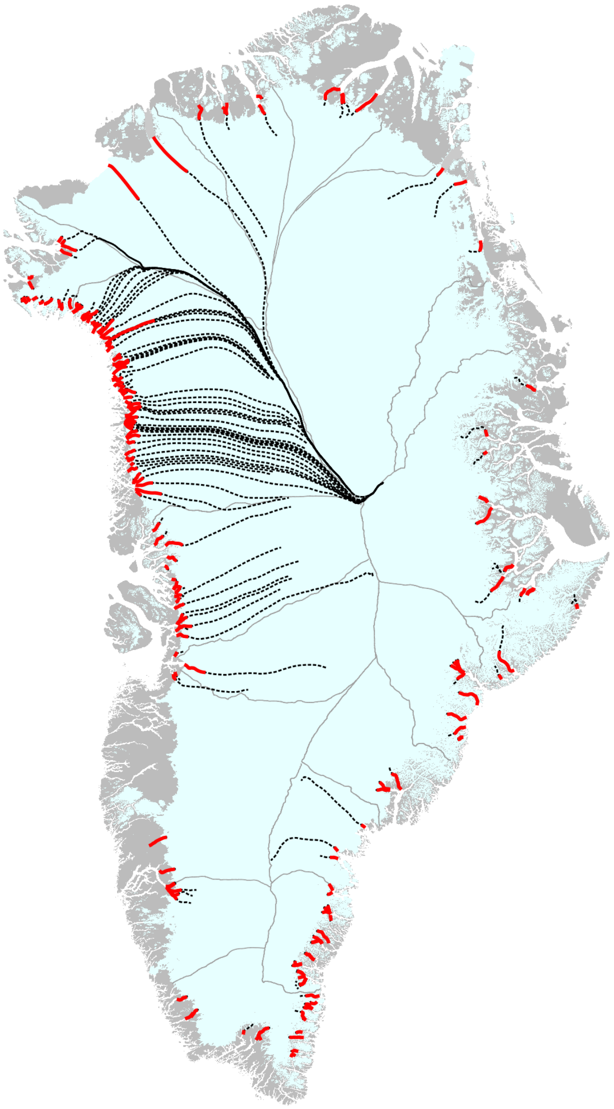

Figure S10. Minimum and maximum glacier-wide thinning limits based on uncertainties in input datasets and uncertainty in the selection of the empirical thinning limit. The red solid line represents the minimum distance from each glacier terminus to the location where Pe=2 among all error realizations. The black dashed line represents the maximum distance from each glacier terminus to the location where Pe=4 among all error realizations.

Table S1. Classification of mountainous and gentle bed topography by drainage system (DS).

DS	min	max	mean	median	std	classification
1.1	-1172.00	1355.00	77.56	19.00	265.40	gentle
1.2	-988.00	1158.00	252.33	231.00	317.58	mountainous
1.3	-476.00	1160.00	362.41	345.00	322.62	mountainous
1.4	-125.00	1011.00	324.97	332.00	200.43	gentle
2.1	-1331.00	1979.00	54.02	-2.00	268.84	gentle
2.2	-409.00	2436.00	813.03	802.00	414.89	mountainous
3.1	-1419.00	2888.00	939.30	902.00	557.63	mountainous
3.2	-715.00	3672.00	1372.08	1455.00	636.66	mountainous
3.3	-1179.00	2978.00	892.86	825.00	586.50	mountainous
4.1	-1111.00	3373.00	551.91	315.00	613.51	mountainous
4.2	-608.00	2050.00	455.07	411.00	256.44	gentle
4.3	-1071.00	2193.00	1057.10	1067.00	360.90	mountainous
5.0	-1157.00	2504.00	1000.00	988.00	370.93	mountainous
6.1	-711.00	1755.00	750.38	724.00	300.06	mountainous
6.2	-687.00	1790.00	288.96	246.00	280.38	gentle
7.1	-1478.00	449.00	-59.02	-53.00	142.09	gentle
7.2	-1085.00	2195.00	185.32	90.00	335.68	mountainous
8.1	-1476.00	1931.00	165.01	156.00	234.15	gentle
8.2	-567.00	1416.00	506.44	538.00	222.42	gentle
